# Expulsion of nuclear DNA to the cytoplasm after viral entry: A mechanism for activation of the cGAS pathway

**DOI:** 10.1073/pnas.2614472123

**Published:** 2026-08-18

**Authors:** Nicolás Romero, Hyung Suk Oh, Max E. Mertens, Maria Ericsson, Kyle N. Stearns, Anne Moscona, David M. Knipe

**Affiliations:** ^a^Department of Microbiology, Blavatnik Institute, Harvard Medical School, Boston, MA 02115; ^b^Electron Microscopy Core, Department of Cell Biology, Harvard Medical School, Boston, MA 02115; ^c^Department of Pediatrics, Columbia University Vagelos College of Physicians and Surgeons, New York, NY 10032; ^d^Center for Host–Pathogen Interaction, Department of Pediatrics, Columbia University Vagelos College of Physicians and Surgeons, New York, NY 10032; ^e^Department of Physiology and Cellular Biophysics, Columbia University Vagelos College of Physicians and Surgeons, New York, NY 10032; ^f^Department of Microbiology and Immunology, Columbia University Vagelos College of Physicians and Surgeons, New York, NY 10032

**Keywords:** innate immunity, viral entry, herpes simplex virus, host response, interferon signaling

## Abstract

Cells respond to viral infection by induction of numerous innate and other host responses to block infection in that cell and surrounding cells. The mechanism(s) by which nuclear replicating DNA viruses and certain RNA viruses elicit responses through the cyclic guanosine monophosphate-adenosine monophosphate synthase (cGAS) cytoplasmic DNA sensor has been elusive. We report here that early events during herpes simplex virus 1 infection induce blebbing of nuclear DNA into the cytoplasm, recruitment of cGAS colocalization with the cytoplasmic DNA, activation of interferon regulatory factor 3, and induction of type 1 human interferon in normal human fibroblasts and keratinocytes. With HSV-1, this is dependent on viral binding and fusion with the surface membrane but independent of transport of the input viral DNA to the nucleus. Infections by herpes simplex virus 2, human cytomegalovirus, or human parainfluenza virus 3 also induce nuclear DNA blebbing, consistent with entry by surface fusion inducing this effect. Extrusion of nuclear DNA through nuclear envelope breaks upon viral entry by fusion represents a mechanism for cells to respond to the physical and/or molecular changes in the infected cell during infection.

## Significance

Cells mount antiviral responses when they detect infection by a virus, and viruses have evolved mechanisms to resist the host responses. In this study, we have discovered a new form of host response. Upon entry of viruses that occurs by fusion of the viral envelope with the plasma membrane, nuclear DNA is released from the nucleus into the cytoplasm, eliciting an interferon antiviral response. Understanding this mechanism could strengthen antiviral immunity or alternatively provide approaches to minimize the host response for beneficial viral infections such as those by viral gene therapy vectors.

Cells respond to viral infection with a variety of antiviral responses, and viruses have evolved mechanisms to evade those responses. Mammalian cells use a network of specialized sensor proteins called pathogen recognition receptors (PRRs) to recognize foreign pathogens or components of those pathogens, collectively termed pathogen-associated molecular patterns (PAMPs), including microbial nucleic acids or other structural components such as lipopolysaccharides and glycoproteins. Upon stimulation, the receptors initiate diverse signaling cascades, including interferons (IFNs) to respond against pathogen infection (1). Interferons induce a broad variety of host antiviral responses that constitute a first-line defense system in mammals against virus infections. Interferons are secreted from virus-infected cells to protect neighboring uninfected cells through the Janus kinase-signal transducer and activator of transcription (JAK-STAT) pathway, in the case of type I interferons through the interferon-α/β receptor (IFNAR). Activation of the JAK-STAT pathway drives expression of interferon-stimulated genes (ISGs), a subset of genes encoding antiviral functions (1). Among these antiviral proteins, the myxovirus resistance 2 gene product, MxB, and the interferon γ-inducible protein 16 (IFI16) are essential in tackling the earliest stages of herpesvirus replication by dismantling entering capsids (2–4), and causing an epigenetic silencing of incoming viral genomes, respectively (5).

Human fibroblasts infected with herpes simplex virus 1 (HSV-1), a human enveloped dsDNA virus from the *Orthoherpesviridae* family (6), mount IFN responses using several receptors including cytoplasmic cyclic guanosine monophosphate-adenosine monophosphate synthase (cGAS), retinoic acid-inducible gene I (RIG-I) and IFI16 (7). Viral gene products then act to evade those responses. The initial IFN response to HSV-1 induces IFNβ gene expression through interferon regulatory factor 3 (IRF3) transcription factor occurs without de novo synthesized viral gene products (8–12).

cGAS, a cytoplasmic dsDNA sensor, is a major factor in the early response to HSV infection, although its ligand, either viral or cellular, remains elusive in human fibroblasts infected with HSV (9, 13). It has been assumed since the discovery of cGAS that its ligand in HSV-infected cells is HSV DNA, leaking either from capsids or the nucleus (14), but the ligand for cGAS in HSV-infected cells remains to be defined.

In the canonical mechanism of cytoplasmic cGAS action, cGAS binds to free dsDNA molecules in the cytoplasm, which activates synthesis of cyclic GMP-AMP (cGAMP), which binds to and activates the stimulator of interferon genes (STING). STING trafficks from the endoplasmic reticulum (ER) to the endoplasmic reticulum-Golgi intermediate compartment (ERGIC) and Golgi apparatus, where TANK-binding kinase 1 (TBK1) and the interferon regulatory factor 3 (IRF3) transcription factor are recruited. Following TBK-1 phosphorylation of IRF3 at Ser386 (15), IRF3 dimerizes and translocates into the nucleus for the expression of IRF3-targeted genes, including the IFNβ gene (1). cGAS has variously been described as being tethered in the cytoplasm (16) or in the nucleus on chromatin (17) until activated. Nuclear cGAS activation has also been reported (18).

The cGAS sensor can also be triggered by the existence of cytoplasmic self-DNA derived from the cell nucleus or mitochondria (reviewed in ref. 19), but also in response to infection with DNA and RNA viruses (reviewed in ref. 20), indicating that viral DNA genomes may not necessarily be the source of cGAS activation, for instance in the SARS CoV2-induced host cell micronuclei formation (21). One paper reported that cGAS was associated with cellular centromeric DNA (18), but this was done with a nonpermissive infection of dendritic cells harvested at 24 hpi with HSV-1, a very late time during a nonproductive infection.

Thus, numerous questions remain for the mechanism(s) of action of cGAS in response to viral infection. How does cytoplasmic cGAS respond to viral DNA usually delivered to the nucleus protected within a capsid? What is the cGAS ligand in virus-infected cells? How does an RNA virus activate cGAS? Does cGAS act in the cytoplasm, nucleus, or both?

While looking for cytoplasmic viral DNA in HSV-1 infected cells, we have made the surprising observation of nuclear DNA blebbing and cytoplasmic DNA spheroids in cells infected with viruses that fuse at the plasma membrane. HSV entry is required but not transport of the viral nucleocapsid to the cell nucleus. Thus, this is a new host response mechanism that can detect either DNA or RNA viruses that enter by fusion at the plasma membrane.

## Results

### Infection with Herpesviruses or a Paramyxovirus Induces the Formation of Cytoplasmic DNA Structures Early After Infection in Normal Human Fibroblasts.

**Fig. 1.**
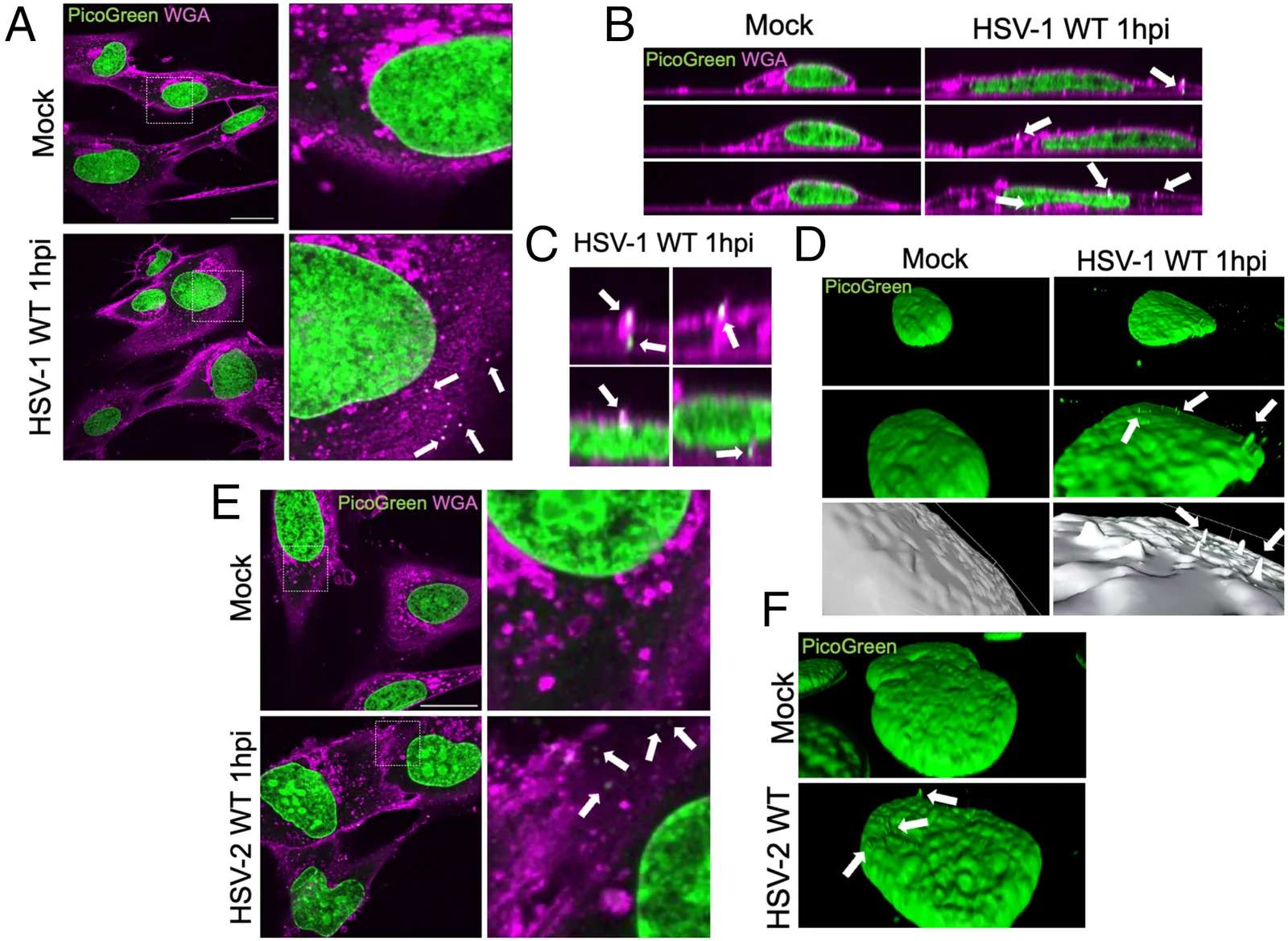
Herpes simplex virus infections induce extranuclear DNA structures shortly upon inoculation. (*A*) HFF cells were mock-infected or infected with wild-type (WT) HSV-1 strain KOS (HSV-1 WT) supernatant stock at an MOI of 5 pfu/cell, fixed at 1 hpi. and stained with the dsDNA fluorescent probe PicoGreen and Texas red-conjugated WGA. Green = PicoGreen; magenta = WGA (n = 4 independent experiments). Expanded views of mock-infected and HSV-1-infected HFF cells are shown. (*B*) Orthogonal views of mock-infected and HSV-1 WT-infected HFF cells at 1 hpi. Green = PicoGreen; magenta = WGA. (*C*) *Inset* images show higher magnification of the orthogonal views presented in (*B*). PicoGreen = green; magenta = Texas-red conjugated WGA. (*D*) Three-dimensional renderings of the PicoGreen fluorescence signal from the apical cell view of mock-infected and HSV-1 WT-infected HFF cells at 1 hpi using the images taken for the experiment shown in (*A*). Volumetric 3-D representations are illustrated in green in the two *Upper* panels. In the *Lower* panel, the apical surface of PicoGreen-stained DNA was modeled and shown in a gray scale. (*E*) HFF cells mock-infected or infected with wild-type HSV-2 strain G (HSV-2 WT) supernatant stock at an MOI of 5 pfu/cell and fixed at 1 hpi. Green = PicoGreen; magenta = Texas red-conjugated WGA. Certain regions of interest showing HSV-2 WT-induced extranuclear DNA were expanded (n = 3 independent experiments). (*F*) Three-dimensional renderings of PicoGreen signal captured from mock-infected and HSV-2 WT-infected HFF cells at 1 hpi. PicoGreen = green. Extranuclear DNA structures are denoted with white arrows. The scale bars in (*A* and *E*) are 20 μm.

Human cells respond very early to infection by HSV-1 by cytoplasmic innate signaling involving STING, cGAS, and IRF-3 activation that induces IFNβ expression (8, 22). To determine if cytoplasmic DNA could be detected in HSV-infected cells, we stained mock- and wild-type HSV-1-infected human foreskin fibroblasts (HFFs) with a highly sensitive dsDNA dye, PicoGreen (23). We observed no cytoplasmic staining due to PicoGreen staining in mock-infected HFF cells but did observe DNA spots or spheroids in the cytoplasm of HSV-1 wild-type-infected HFF as early as 1 h postinfection (hpi) (Fig. 1 *A*–*C*). Using spinning disk confocal microscopy, we observed cytoplasmic PicoGreen-labeled dots within the cellular contour and near the edge of the cytoplasm, as defined by staining with fluorescently labeled wheat germ agglutinin (WGA) in the x-y plane (Fig. 1*A*). Furthermore, z axis views of infected HFF cells showed extranuclear DNA emerging or blebbing from the apical surface of the cell nucleus and extranuclear DNA located relatively distant from the cell nucleus (Fig. 1 *B* and *C*). At least one nuclear bleb or cytoplasmic DNA spheroid was found in about 70% of the infected cells (*SI Appendix*, Fig. S1*A*) with an average of three cytoplasmic DNA structures per cell. In three-dimensional renderings of the PicoGreen fluorescence signal, we further observed dramatic blebbing of DNA from the cell nucleus surface (Fig. 1*D*).

**Fig. 2.**
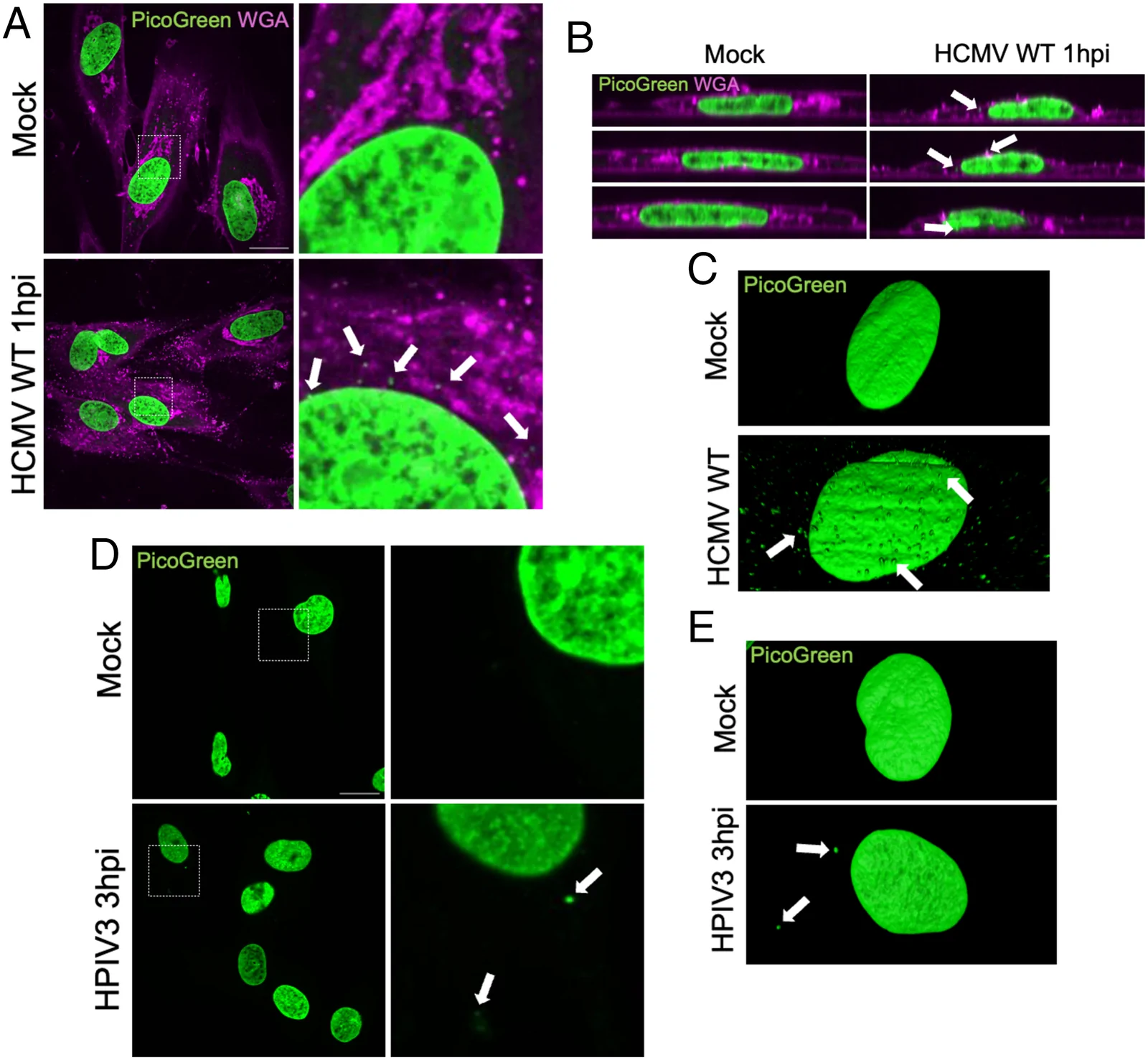
HCMV and HPIV3 infections trigger similar extranuclear DNA structures. (*A*) HFF cells were mock-infected or infected with HCMV strain AD169 (HCMV WT) supernatant stock at an MOI of 5 and fixed at 1 hpi (n = 3 independent experiments). Cells were stained with PicoGreen (green) and Texas red-conjugated WGA (magenta). *Inset* images show increased magnification of PicoGreen-stained cytoplasmic DNA. (*B*) Orthogonal views of mock-infected and HCMV WT-infected HFF cells at 1 hpi. Green = PicoGreen; magenta = Texas red-conjugated WGA. (*C*) Three-dimensional rendering of the PicoGreen fluorescence signal in mock-infected HCMV WT-infected HFF cells at 1 hpi, from images corresponding to the experiment shown in (*A*). PicoGreen = green. (*D*) HFF cells were mock-infected or infected with HPIV3 at an MOI of 1, fixed at 3 hpi, and stained with PicoGreen dye (n = 3 independent experiments). (*E*) Three-dimensional images of PicoGreen signal corresponding to mock-infected and HPIV3-infected HFF cells at 3 hpi, presented from an apical view. PicoGreen = green. White arrows denote PicoGreen-labeled extranuclear DNA. The scale bars in (*A* and *D*) are 20 µm.

We also observed cytoplasmic DNA in HSV-2 wild-type-infected HFF cells (Fig. 1 *E* and *F* and *SI Appendix*, Fig. S1*B*) and in fibroblasts infected with another herpesvirus, human cytomegalovirus (HCMV) (Fig. 2 *A*–*C* and *SI Appendix*, Fig. S1*C*), showing that the phenotype was observed with at least two subfamilies of herpesviruses. In a test of another virus that fuses at the plasma membrane for entry, we observed that HFF cells infected with an enveloped RNA virus, human parainfluenza virus 3 (HPIV3), showed PicoGreen-stained cytoplasmic DNA at 3 hpi (Fig. 2*D* and *SI Appendix*, Fig. S1*D*), although to a lesser extent than in herpesviral infections. No cytoplasmic DNA was observed in the cytoplasm of HFF cells infected with vesicular stomatitis virus (VSV) (*SI Appendix*, Fig. S1 *E*–*G*), although the VSV-GFP-infected HFF cells expressed GFP (*SI Appendix*, Fig. S1*H*). Because VSV enters cells by endocytosis, these results argued that the trigger for nuclear DNA blebbing might be viral fusion at the plasma membrane.

### Viral Attachment and Penetration, But Not Viral Gene Expression, Are Needed to Form Cytoplasmic DNA Structures.

**Fig. 3.**
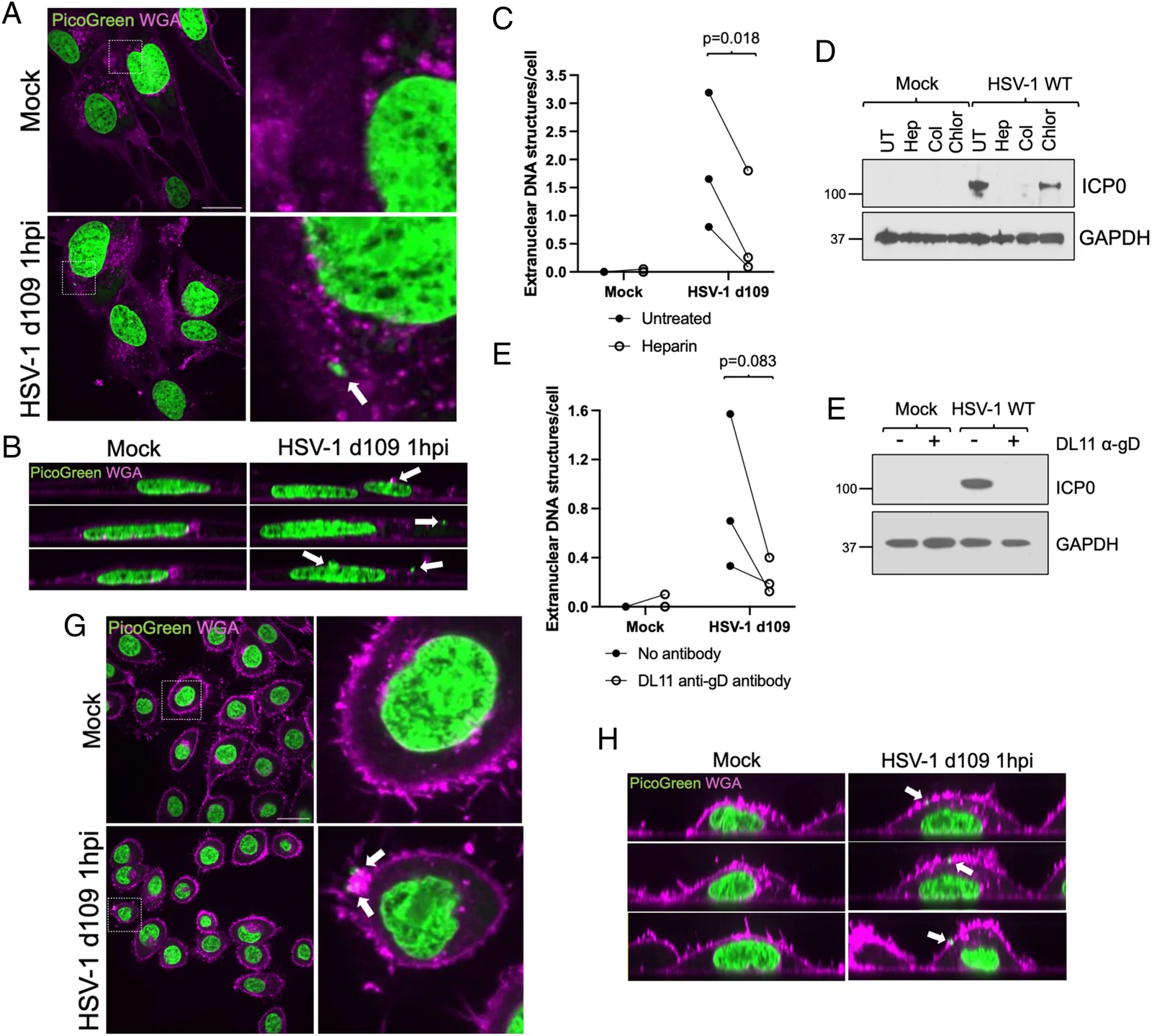
HSV-1 gene expression is not required for the formation of extranuclear DNA structures. (*A*) HFF cells were mock-infected or infected with HSV-1 *d*109 virus at an MOI of 5, fixed at 1 hpi, and stained with PicoGreen (green) and Texas red-conjugated WGA (magenta) (n = 3 independent experiments). *Insets* show magnified images. (*B*) Orthogonal views of mock-infected and HSV-1 *d*109-infected HFF cells at 1 hpi using the images taken for the experiment shown in (*A*). Green = PicoGreen; magenta = Texas red-conjugated WGA. Extranuclear DNA structures are denoted with white arrows in (*A* and *B*). (*C*) HFF cells mock-infected or infected with an MOI of 5 pfu/cell of HSV-1 *d*109 at MOI = 5 in the presence or absence of heparin, which was added to cells 1 h before infection and was maintained during infection. Cells were fixed at 1 hpi and stained with PicoGreen (green) and Texas red-conjugated WGA (magenta) (n = 3 independent experiments). The graph represents the individual values for the number of extranuclear DNA structures determined in the *Y*/*Z* axes per cell. The values for the same biological experiment are connected to show the trend in mock-infected or HSV-1 *d*109-infected HFF cells in the presence or absence of heparin. *P* = 0.018 using a paired one-tailed Student’s *t* test. (*D*) Western blotting analysis of HSV-1 ICP0 protein expression in HFF cells mock-infected or infected with HSV-1 WT at an MOI of 5 pfu/cell for 3 hpi in the presence or absence of heparin (Hep), colchicine (Col), or chloroquine (Chlor). UT stands for untreated. (n = 3 independent experiments). Heparin, colchicine, chloroquine, or the appropriate control solutions were added to the cell cultures 1 h before infection and kept during infection. (*E*) HFF cells were mock-infected or infected with 10^5^ pfu of HSV-1 *d*109 or the same amount of virus incubated with 5 μg/mL of the HSV-1-neutralizing anti-gD DL11 antibody for 16 h at 4 °C. Cells were fixed at 1 hpi and stained with PicoGreen and Texas red-conjugated WGA (n = 3 independent experiments). The graph represents the individual values of the extranuclear DNA structures/cell counted across the Y/Z axes. *P* = 0.083 with a paired one-tailed Student’s *t* test. (*F*) Immunoblot for HSV-1 ICP0 protein in HFF cells mock-infected or infected with 10^5^ pfu of WT HSV-1 KOS at 6 hpi, previously incubated or not for 16 h at 4 °C with 5 μg/mL of the HSV-1-neutralizing anti-gD DL11 antibody. (n = 3 independent experiments). HSV-1 ICP0 protein levels were normalized to GAPDH protein. (*G*) NOK cells were mock-infected or infected at MOI of 5 pfu/cell with the HSV-1 *d*109 supernatant stock, fixed at 1 hpi, and stained with PicoGreen for dsDNA labeling and Texas red-conjugated WGA for cell membrane staining (n = 3 independent experiments). Green = PicoGreen; magenta = Texas red-conjugated WGA. *Inset* images show magnified regions. (*H*) Orthogonal representation of mock or HSV-1 *d*109-infected NOK cells, from images of the experiment shown in (*G*). Green = PicoGreen; magenta = Texas red-conjugated WGA. White arrows denote DNA blebs in (*G* and *H*). The scale bars in (*A* and *G*) are 20 μm. Protein markers in (*D* and *F*) are expressed in kilodaltons.

To determine the role of HSV-1 gene expression in nuclear DNA blebbing, we infected HFF cells with the HSV-1 *d*109 mutant virus, which has all five IE genes deleted and thus does not express any viral gene products de novo (24). We observed that cells infected with the HSV-1 *d*109 mutant virus produced phenotypically similar extranuclear DNA structures to those in wild-type HSV-1 infection (Fig. 3 *A* and *B* and *SI Appendix*, Fig. S1 *I* and *J*), demonstrating that cytoplasmic DNA formation was independent of viral gene expression.

To further characterize the cytoplasmic DNA induced by *d*109 virus in the absence of viral gene expression and to examine potential mitochondrial DNA in the cytoplasm, we generated overexposed images of HFF cells that were mock-infected or infected with *d*109 virus and then stained with PicoGreen and Mitotracker, which accumulates in mitochondria due to their membrane potential, at 3 hpi. In the overexposed images of mock-infected cells, we observed PicoGreen diffuse staining and small puncta, and some of the small puncta were within the Mitotracker-stained structures (*SI Appendix*, Fig. S3*A*). However, at the same exposure, the infected cell cytoplasm was almost entirely green with larger Picogreen blobs, arguing that the apparently larger amount of cytoplasmic DNA in infected cells did not come from mitochondrial DNA (*SI Appendix*, Fig. S3*A*).

To determine if viral attachment was needed for nuclear DNA blebbing, we inhibited viral attachment to its initial cellular binding sites, heparan proteoglycans (25), by performing infection in the presence of heparin. Heparin treatment significantly reduced the number of extranuclear DNA structures identified in HSV-1 *d*109–infected HFF cells at 1 hpi (Fig. 3*C*). We confirmed that heparin blocked expression of the IE ICP0 protein in wild-type HSV-1-infected HFF cells at 3 hpi, to the same extent as the microtubule inhibitor colchicine, which prevents capsid transport to the nuclear pores (Fig. 3*D*) (26). We observed ICP0 protein accumulation in the presence of the endocytosis inhibitor chloroquine (Fig. 3*D*), arguing that HSV-1 entry in HFF cells is at least partly mediated by fusion of the virus envelope with the plasma membrane.

To further determine the role of HSV-1 entry in DNA blebbing, we examined glycoprotein D (gD)-based entry and its role in DNA blebbing. We observed that incubation with the neutralizing anti-HSV-1 gD-specific DL11 monoclonal antibody, which prevents the binding of gD protein to cellular receptors nectin-1 or HVEM (27) also reduced nuclear DNA blebbing at 1 hpi (Fig. 3*E*). The neutralizing activity of the DL11 antibody was verified in wild-type HSV-1 infection, as it reduced ICP0 expression at 6 hpi (Fig. 3*F*). In total, these results argued that viral attachment and entry were needed for induction of nuclear DNA blebbing. The antibody results further argued that free DNA was not causing the effect, and this was confirmed by a lack of an effect on blebbing when we treated the HSV-1 inoculum with DNase.

The nuclear DNA blebbing response was also observed in HSV-1 *d*109–infected normal oral keratinocytes (NOK cells) (Fig. 3 *G* and *H* and *SI Appendix*, Fig. S1*K*), indicating that this response to HSV-1 entry occurs in human epithelial cells. These specific results showed that nuclear DNA blebbing in HSV-1-infected fibroblasts and keratinocytes cells is associated with virus entry but does not require viral gene expression.

### Ultrastructural Basis of Nuclear DNA Blebbing.

**Fig. 4.**
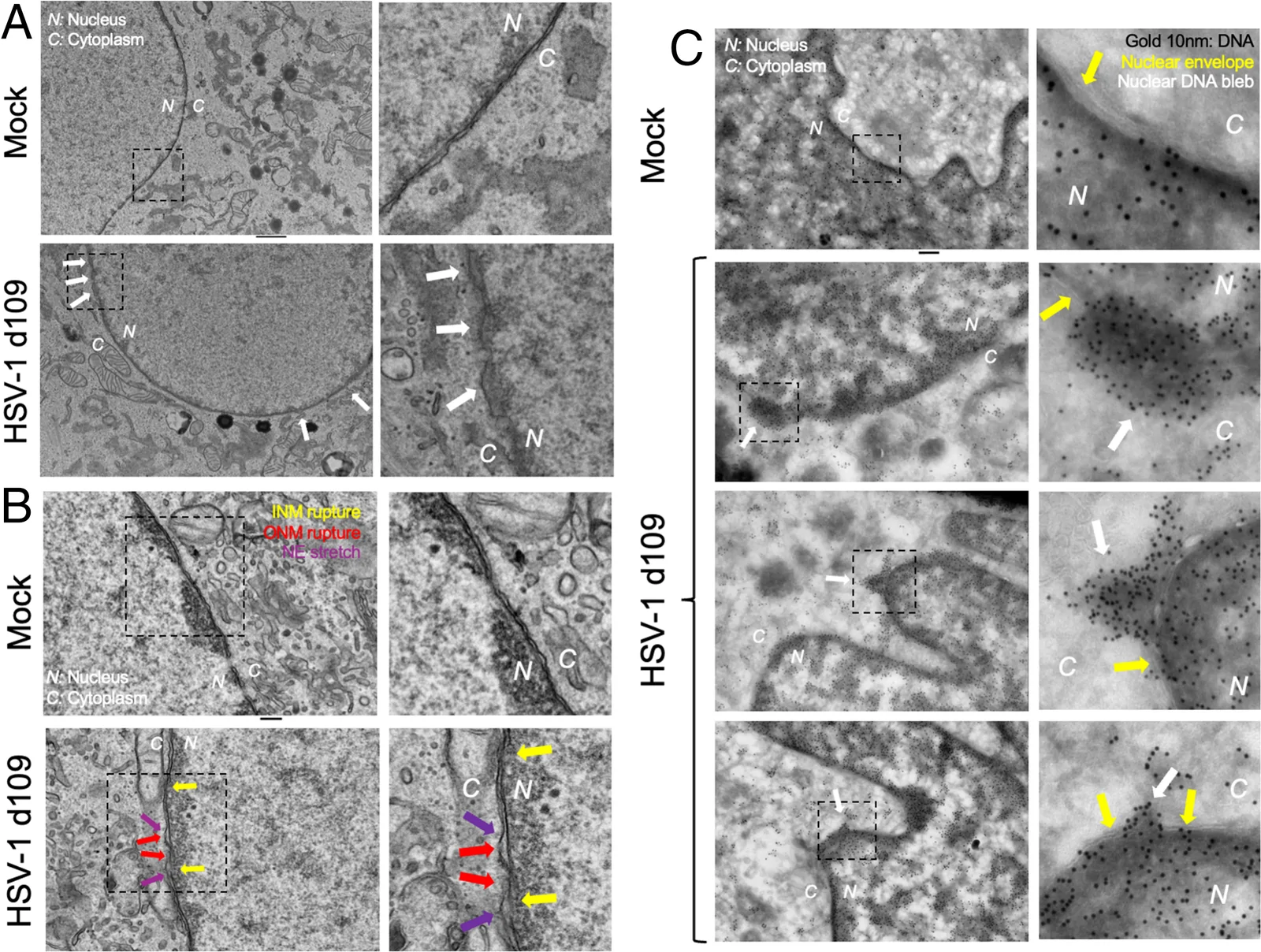
Ultrastructural characterization of nuclear DNA blebs by TEM. (*A*) Positive staining in mock-infected and HSV-1 *d*109-infected HFF cells at 3 hpi (MOI = 5 pfu/cell) visualized by transmission electron microscopy (TEM). White arrows point to nuclear envelope membrane separations. Direct magnification was 4,800×, where one pixel is 1.46 nm. The scale bar represents 1 μm. (n = 2 independent experiments). (*B*) Representative examples of mock-infected and HSV-1 *d*109-infected HFF cells at 3 hpi (MOI = 5 pfu/cell), which were subject to positive staining, serial ultrathin sectioning, and TEM imaging. Nuclear envelope ruptures and separations are denoted with arrows: yellow = inner nuclear membrane (INM) rupture; red = outer nuclear membrane (ONM) rupture; purple = nuclear envelope (NE) separation. Direct magnification was 20,000×, and a pixel is 0.9 nm. The scale bar is 200 nm. (*C*) Immuno-gold labeling of DNA in mock-infected or HSV-1 *d*109-infected HFF cells, using the Tokuyasu method. Gold nanoparticles (10 nm) label dsDNA. White arrows denote nuclear DNA blebs streaming through the nuclear envelope. Yellow arrows indicate the double nuclear membrane. Direct magnification was 20,000×, and one pixel corresponds to 0.9 nm. The scale bar is 200 nm. In every image of the figure, *N* = nucleus, and *C* = cytoplasm.

To define the ultrastructure of the nuclear envelope during blebbing, we utilized transmission electron microscopy to examine ultrathin sections of mock-infected and HSV-1 *d*109–infected HFF cells at 3 hpi. HSV-1 *d*109–infected cells exhibited increased separation between the inner and outer nuclear membranes at discrete regions of the nuclear envelope, compared to mock-infected cells (Fig. 4*A*). These regions showed a higher frequency of membrane stretches ≥60 nm and ≥100 nm compared to mock-infected cells (*SI Appendix*, Fig. S2 *A* and *B*). The outer nuclear membrane was consistently the more distended membrane, extending toward the cytoplasm (Fig. 4*A*). Serial ultrathin sections further revealed apparent breaches in the nuclear envelope, involving both the inner and outer nuclear membranes, often adjacent to stretched nuclear envelope sites (Fig. 4*B*). In contrast, nuclear pore number and size were comparable between HSV-1 *d*109–infected and uninfected cells (*SI Appendix*, Fig. S2 *C* and *D*). Most conclusively, immuno-gold labeling of DNA revealed DNA exiting the nucleus through breaks in the double nuclear membrane (Fig. 4*C*). Notably, these nuclear DNA blebs were absent in mock-infected HFF cells (Fig. 4*C*). Overall, the TEM findings confirmed the DNA blebbing observed by light microscopy and supported nuclear envelope rupture as the underlying cause of nuclear DNA blebbing.

### Cytoplasmic DNA Structures Colocalize with cGAS Aggregates.

**Fig. 5.**
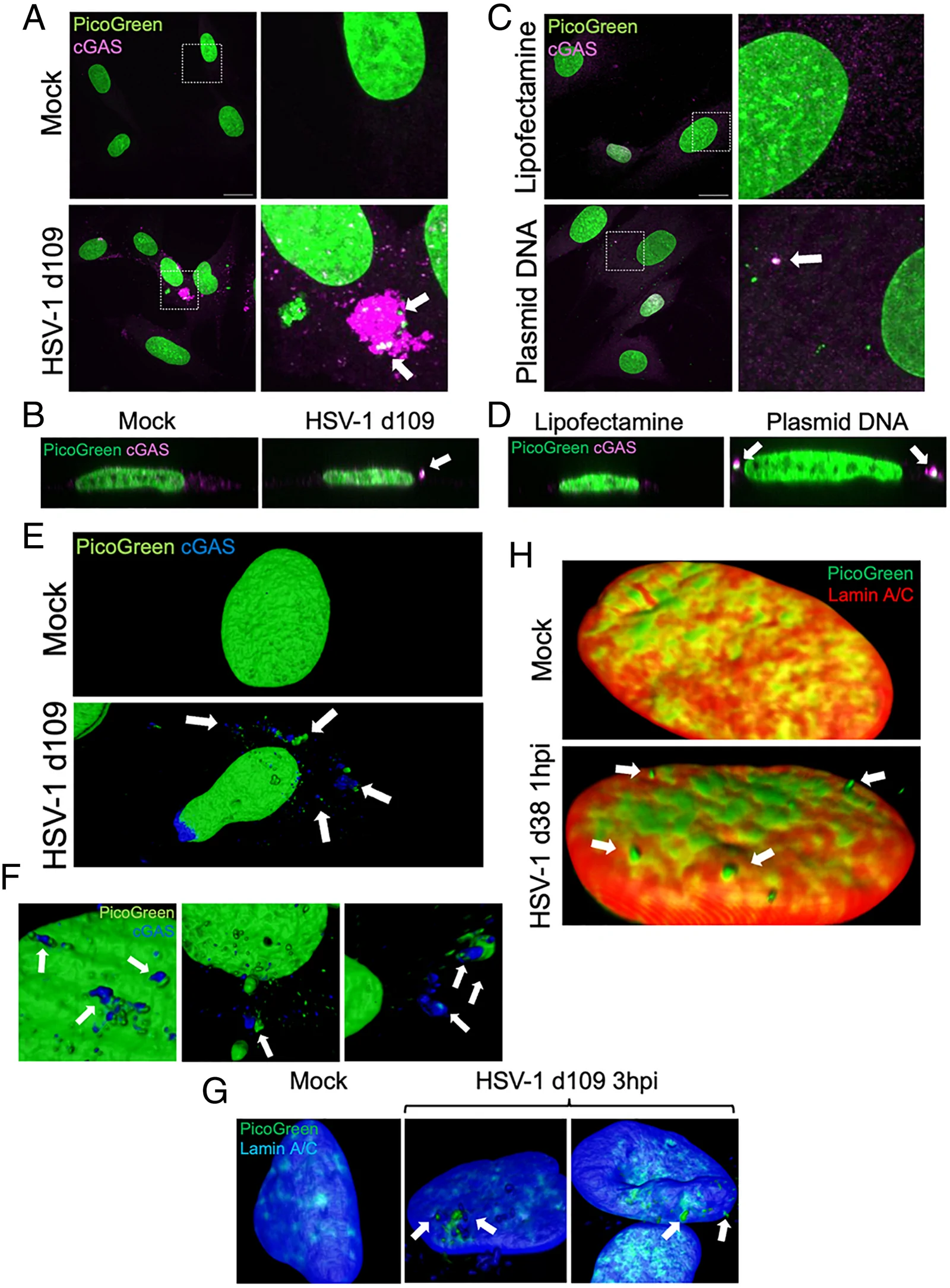
Colocalization of cGAS with virus-induced nuclear DNA blebs. (*A*) HFF cells were mock-infected or infected with HSV-1 *d*109 virus at MOI = 5, fixed at 3 hpi (n = 3 independent experiments), and stained with PicoGreen and anti-cGAS antibody. Green = PicoGreen; magenta = cGAS. *Inset* images show magnified regions where the cGAS-DNA foci were observed. Z-projections are presented. (*B*) Orthogonal views of mock-infected and HSV-1 *d*109-infected HFF cells at 3 hpi, corresponding to the experimental set-up shown in (*A*). PicoGreen = green; cGAS = magenta. White arrows denote HSV-1 *d*109-induced cGAS-DNA foci in (*A* and *B*). (*C*) HFF cells transfected with 200 ng of pUC19 plasmid with lipofectamine or only treated with lipofectamine (transfection control) and were fixed 3 h later (n = 2 independent experiments). Cells were stained with PicoGreen and anti-cGAS antibody. Green = PicoGreen; magenta = cGAS. The confocal micrographs are presented as Z-projections. Areas showing transfected plasmid DNA-cGAS foci are magnified. (*D*) Orthogonal views of HFF cells transfected or not (lipofectamine = transfection control) with plasmid DNA for 3 h, using the experimental set-up shown in (*C*). PicoGreen = green; cGAS = magenta. White arrows denote plasmid DNA-cGAS foci in (*C* and *D*). (*E*) Three-dimensional renderings of PicoGreen-stained DNA (green) and cGAS (blue) in mock- or *d*109-infected HFF cells fixed at 3 hpi, using the same experimental set-up as in (*A* and *B*). Cells were stained with PicoGreen and anti-cGAS antibody. (*F*) *Inset* images of the 3-D rendered the HSV-1 *d*109-infected HFF cells for improved visualization of the cGAS (blue)-DNA (green) foci. White arrows denote 3-D cytoplasmic DNA-cGAS foci in (*E* and *F*). (*G*) 3-D rendering of PicoGreen signal (green) and lamin A/C protein (blue) in mock- and HSV-1 *d*109-infected HFF cells fixed at 3 hpi (n = 3 independent experiments). (*H*) Three-dimensional renderings of mock- and HSV-1 *d*38-infected HFF cells (MOI of 5 pfu/cell) fixed 1 hpi and stained PicoGreen and anti-lamin A/C antibody. PicoGreen = green and lamin A/C = red. White arrows denote nuclear DNA blebs in (*G* and *H*). The scale bars are 20 µm in (*A* and *C*).

We observed previously that IFI16 and cGAS cellular DNA sensors can both act and even cooperate to promote type I interferon production in HFF cells responding to HSV-1 infection (9). When we performed immunofluorescence to determine the localization of cGAS in HSV-1 *d*109–infected HFF cells, we observed aggregates of the cytoplasmic DNA sensor cGAS localizing at or near Picogreen-labeled HSV-1 *d*109-induced nuclear DNA blebs at 3 hpi in HFF cells, but not in mock-infected HFF cells (Fig. 5 *A* and *B*). Furthermore, the numbers of cytoplasmic cGAS foci per cell were increased significantly in HSV-1 *d*109 infected cells (*SI Appendix*, Fig. S2*G*). In contrast, IFI16 was entirely restricted to the cell nucleus (*SI Appendix*, Fig. S2 *E* and *F*).

Cytoplasmic cGAS colocalized with transfected plasmid DNA, as shown previously (9), and these foci were not detected in lipofectamine carrier-treated cells (Fig. 5 *C* and *D*). The association of cGAS aggregates with nuclear DNA blebs was better visualized using 3-dimensional rendering images (Fig. 5 *E* and *F*), confirming the physical proximity of the HSV-1 *d*109-induced cytoplasmic DNA and cGAS. Colocalization was determined between cGAS and cytoplasmic DNA (*SI Appendix*, Fig. S2*H*). As a control, we measured colocalization of the ICP5 capsid protein building with cytoplasmic and found that ICP5 shows less colocalization with cytoplasmic DNA than cGAS (*SI Appendix*, Fig. S2 *H* and *J*), arguing for at least partial specific colocalization of cGAS and cytoplasmic DNA. Thus, the cGAS aggregates around cytoplasmic DNA potentially constitute hubs for cGAS-DNA interaction, triggering cGAMP synthesis and downstream activation of STING.

Dual staining of nuclear lamin A/C and dsDNA showed that many of the DNA blebs appeared to bud through the nuclear lamina in HSV-1 *d*109–infected cells at 3hpi (Fig. 5*G*) or in HSV-1 *d*38 infection at 1hpi, a mutant virus that exhibited enhanced ability to induce DNA blebbing (Fig. 5*H*), arguing that the blebs are covered with little of the nuclear lamina. HSV-1 *d*109–infected NOK cells also exhibited cytoplasmic cGAS aggregates at 3 hpi (*SI Appendix*, Fig. S2 *K* and *L*), indicating that this cytoplasmic DNA response was also mounted by epithelial cells and is not cell type–specific.

We further tested the functional requirements for formation of cytoplasmic cGAS aggregates. Heparin treatment, which reduces HSV-1 binding to cells, blocked the formation of PicoGreen/cGAS foci in HSV-1 *d*109–infected HFF cells at 3 hpi (*SI Appendix*, Fig. S3*B*). In agreement with HPIV3-induced nuclear DNA blebbing exemplified in Fig. 2 *D* and *E*, HPIV3-infected HFF cells at 3 hpi exhibited cGAS aggregates overlapping with PicoGreen-labeled cytoplasmic DNA structures comparable to those found in HSV-1 *d*109–infected HFF cells (*SI Appendix*, Fig. S3*C*). Similarly, cGAS accumulation and cytoplasmic DNA-cGAS foci were observed in HCMV-infected HFF cells at 3hpi (*SI Appendix*, Fig. S3*D*). These observations supported the idea that fusion of the viral envelope with the plasma membrane triggers the expulsion of nuclear DNA to the cytoplasm with either DNA or RNA viruses that fuse at the plasma membrane. Nevertheless, viral-independent polyethylene glycol-driven cell–cell fusion did not trigger nuclear DNA blebbing (*SI Appendix*, Fig. S3*E*), indicating that the nuclear DNA blebbing response requires viral fusogen protein activity at the plasma membrane for triggering.

### Nuclear DNA Blebbing Occurs Independently of Viral Genome Transport to and Uncoating at the Cell Nucleus.

**Fig. 6.**
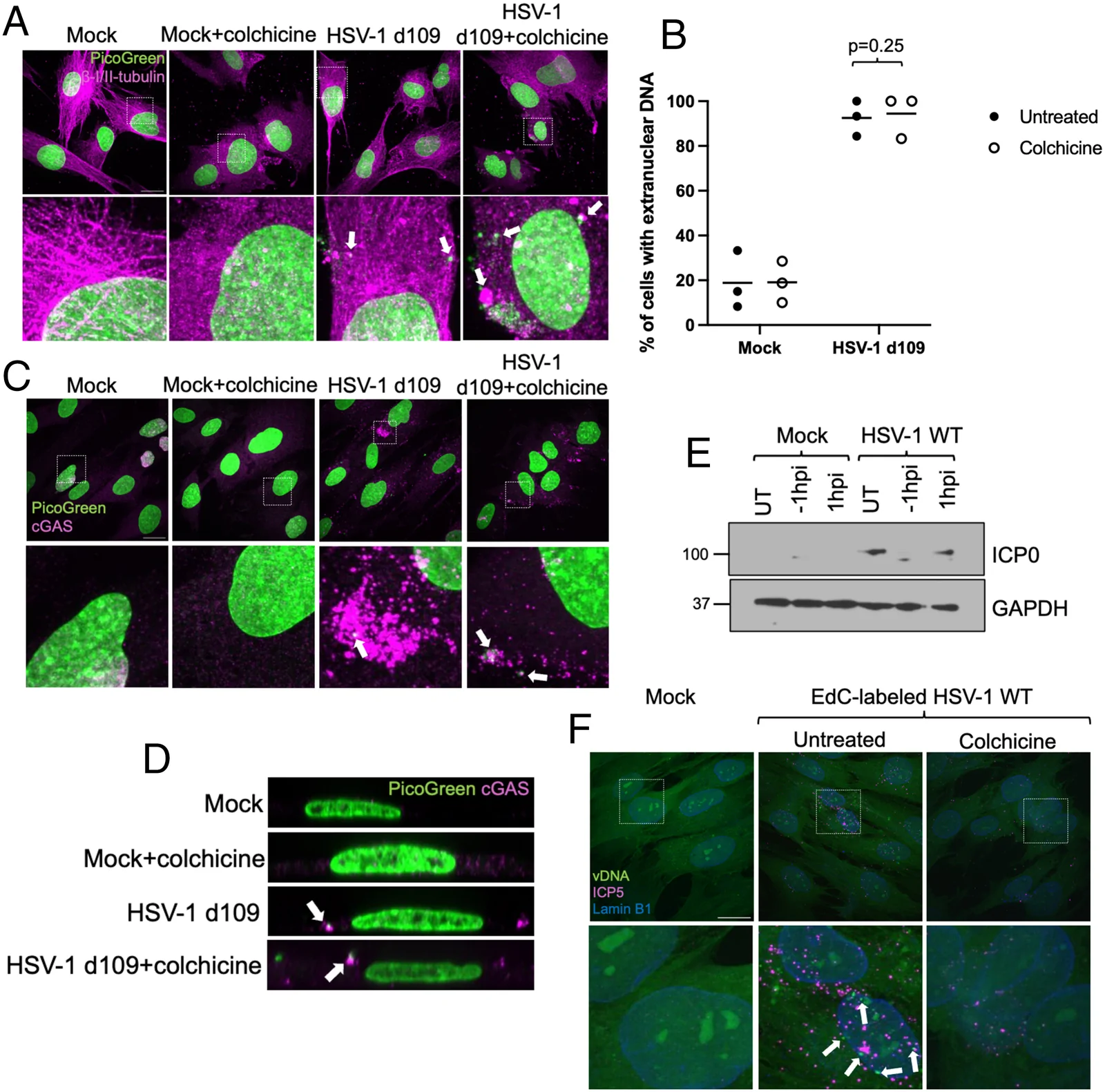
Microtubule-dependent transport of nucleocapsids to nuclear pores and viral genome delivery into cell nuclei are not required for nuclear DNA blebbing. (*A*) HFF cells were mock-infected or infected with HSV-1 *d*109 virus an MOI of 5 pfu/cell in the presence or absence of colchicine, which was added to cells 1 h before infection and kept throughout infection. Cells were fixed at 3 hpi, stained with PicoGreen and anti-β-I/II-tubulin antibody. Z-projection micrographs are shown. Green = PicoGreen, magenta = β-I/II-tubulin (n = 3 independent experiments). (*B*) The mean and individual measurements of the percentage of mock-infected and HSV-1 *d*109-infected HFF cells at 3 hpi (MOI = 5) with extranuclear DNA structures stained by PicoGreen in the presence or absence of colchicine are shown (n = 3 independent experiments). A one-tailed paired Student’s *t* test revealed no statistical significance (*P* = 0.25). (*C*) As in (*A*), the fixed cells were stained with anti-cGAS antibody (magenta) and Picogreen (green) (n = 3 independent experiments). The images are shown as Z-projections. *Inset* images present magnified regions where cytoplasmic DNA-cGAS foci are found. (*D*) Using the images from the experiment shown in (*C*), orthogonal views were generated. Green = PicoGreen; magenta = cGAS protein. (*E*) Western blot analysis of ICP0 protein expression in HFF cells mock-infected or infected with HSV-1 WT at an MOI of 5 in the presence or absence of colchicine, added before, during, and after virus inoculation (at −1 hpi), or only immediately after inoculation (at 1 hpi) (n = 3 independent experiments). UT = untreated conditions. Protein markers are expressed in kilodaltons. (*F*) HFF cells were pretreated or not with colchicine for 1 h before infection with EdC-labeled HSV-1 WT virus stock an MOI of 20 pfu/cell and fixed at 1 hpi (n = 2 independent experiments). Colchicine treatment was maintained during virus adsorption. The input EdC-labeled HSV-1 genomes were detected using a copper-catalyzed click chemistry reaction. *Inset* images show EdC-labeled viral genomes, denoted with white arrows. Immunolabeling of the lamin B1 protein defined the cell nucleus boundaries. Z-projections are shown. Green = EdC-labeled viral DNA, blue = lamin B1, and magenta = HSV-1 ICP5. The scale bar in (*A*, *C*, and *F*) is 20 µm.

To determine if the transport of the incoming HSV-1 DNA to the nucleus was needed for nuclear DNA blebbing, we treated cells with colchicine to disrupt microtubules, which are essential for transport of capsids to the nuclear pores (26). We observed that colchicine treatment disrupted beta-I/II-tubulin filaments in mock-infected or infected cells (Fig. 6*A*) but did not significantly impact the appearance of PicoGreen-labeled cytoplasmic DNA in HSV-1 *d*109–infected HFF cells at 3 hpi (Fig. 6 *A* and *B*) or prevent the formation of cGAS condensates colocalizing with PicoGreen-stained DNA blebs in HSV-1 *d*109 infection at 3 hpi (Fig. 6 *C* and *D*). Importantly, colchicine treatment reduced ICP0 accumulation in wild-type HSV-1 infection at 3 hpi when added 1 h before infection, but not when added 1 h after HSV-1 infection, confirming that colchicine treatment blocked the trafficking of capsids to the nucleus, but did not intrinsically reduce ICP0 protein expression (Fig. 6*E*).

Furthermore, we observed that colchicine treatment reduced the uncoating of 5-ethynyl-2′-deoxycytidine (EdC)-labeled viral genomes in wild-type HSV-1-infected HFF cells at 1 hpi (Fig. 6*F*), despite exhibiting similar input capsids in the cell cytoplasm. Mock-infected HFF cells were negative for ICP5 and EdC-labeled viral genomes (Fig. 6*F*). Collectively, these results argued that the transport of capsids to the nucleus and the genome de-envelopment at the nuclear pores were not essential for the nuclear DNA blebbing phenomenon in response to HSV-1 entry.

To determine if binding was sufficient for induction of cGAS signaling, we allowed HSV-1 *d*109 virus to bind to cells for 1 h on ice and then incubated the cells at 37 °C for 10, 20, or 60 min before treating the cells with low pH buffer to inactivate extracellular virus and then harvesting at 3 hpi and staining with PicoGreen and anti-cGAS antibody to examine their cytoplasmic localization. We observed little cytoplasmic localization with 10 or 20 min of incubation but increased cytoplasmic staining with 60 min of incubation, arguing that binding was not sufficient and some time for entry, at least 20 to 60 min, was required (*SI Appendix*, Fig. S3*F*).

### Nuclear DNA Blebbing Primes cGAS-Dependent Activation of IRF3 in HSV-1 *d*109–Infected HFF Cells.

**Fig. 7.**
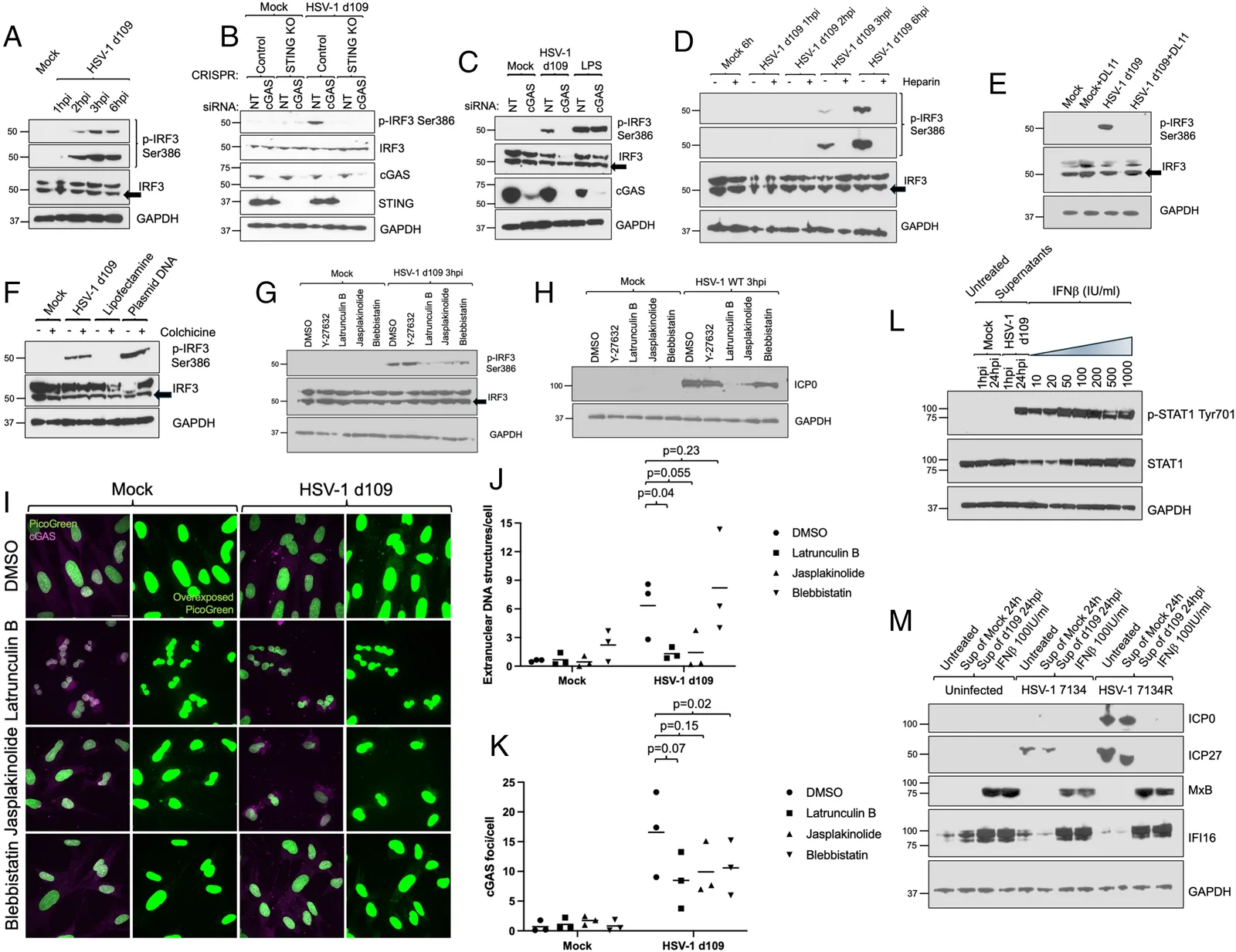
HSV-1 *d*109-induced nuclear DNA blebbing activates type I interferon expression. (*A*) Western blot analysis of pIRF3 Ser386 in HFF cells mock-infected or infected with HSV-1 *d*109 mutant at an MOI of 20, with harvest at 1 to 6 hpi (n = 3 independent experiments). Two different exposures are shown. (*B*) Western blot analysis of pIRF3 in CRISPR Cas9 control or CRISPR Cas9 STING KO HFF cells transfected with either nontarget (NT) or cGAS-specific siRNAs for 48 h and then mock-infected or infected with HSV-1 *d*109 at MOI of 20, and cell lysates were harvested at 3 hpi (n = 3 independent experiments). (*C*) Western blot analysis of pIRF3 was in HFF cells transfected with nontarget (NT) siRNAs or cGAS-specific siRNAs, for 48 h after siRNA transfection, or mock-infected or infected with HSV-1 *d*109 at MOI = 20. LPS lanes contain HFF cells with a 1-h treatment with *Escherichia coli* lipopolysaccharides (LPS) (n = 3 independent experiments). (*D*) Western blot analysis of pIRF3 in HFF cells pretreated or not with heparin for 1 h and then mock-infected or infected with HSV-1 *d*109 at MOI = 20 and harvested at 1 to 6 hpi (n = 3 independent experiments). Heparin was also added during virus adsorption and maintained throughout the infection. Two exposures of the blot are shown. (*E*) Western blot analysis of pIRF3 in HFF cells mock-infected or infected with 10^5^ pfu of HSV-*d*109 that had been incubated or not for 16 h at 4 °C with 5 μg/mL of the HSV-1-neutralizing anti-gD DL11 antibody and harvested at 6 hpi (n = 3 independent experiments). (*F*) Western blot analysis of pIRF3 in HFF cells mock-infected or infected with HSV-1 *d*109 at MOI = 20 or transfected or not (lipofectamine) with 200 ng of pUC19 plasmid for 6 h. These conditions were tested in the presence or absence of colchicine, added to cell cultures 1 h before infection or transfection, and maintained until cell harvest (n = 3 independent experiments). (*G*) HFF cells were pretreated for 1 h with Y-27632, latrunculin B, jasplakinolide, or blebbistatin, and then mock-infected or infected with HSV-1 *d*109 at an MOI of 20 pfu/cell, and cell lysates were harvested at 3 hpi (n = 3 independent experiments). DMSO treatment was used as a drug solvent control. Treatments were kept during virus adsorption and throughout the infection. Shown is a western blot for pIRF3. (*H*) As in (*G*), pretreated cells were either infected or not with HSV-1 WT for 3 h at an MOI of 5 pfu/cell (n = 3 independent experiments). Shown is a western blot for HSV-1 ICP0 protein. (*I*) HFF cells were pretreated for 1 h with DMSO, latrunculin B, jasplakinolide, or blebbistatin, followed by infection or not with HSV-1 *d*109 (MOI = 5 pfu/cell) for 3 h (n = 3 independent experiments). Drugs or vehicle remained in the cell cultures during virus adsorption and throughout the infection. Cells were stained with anti-cGAS and PicoGreen, and Z-projections are shown. PicoGreen = green and cGAS = magenta. Overexposed PicoGreen images are illustrated to visualize tinier nuclear DNA blebs. The scale bar is 20 μm. Values shown are individual and mean values for extranuclear DNA structures/cell (*J*) and cGAS foci/cell (*K*) using images from the experiment shown in (*I*). Individual one-tailed paired Student’s tests were conducted to independently compare values for HSV-1 *d*109–infected cells treated with DMSO to those treated with latrunculin B, jasplakinolide, or blebbistatin. (*L*) Western blot analysis of p-STAT1 T701 in new HFF cells treated for 30 min with 10 to 1,000 IU/mL of human recombinant purified interferon beta (IFNβ), or with supernatants collected from mock-infected or HSV-1 *d*109–infected HFF cells at 1 and 24 hpi, after their infection or not with HSV-1 *d*109 with an MOI = 20 (n = 3 independent experiments). (*M*) HFF cells were incubated or not for 20 h with supernatant collected from mock-infected or HSV-1 *d*109–infected HFF cells at 24 hpi, or with 100 IU/mL recombinant purified IFNβ, and then, infected or not with replication competent ICP0- and ICP0+ HSV-1 strains (HSV-1 7134 and HSV-1 7134R respectively, MOI = 1) for 6 h. Shown is a western blot to detect HSV-1 IE ICP0 and ICP27, or ISG proteins MxB and IFI16 (n = 3 independent experiments). In western blot assays, total IRF3 immunoblots normalized phosphorylated IRF3 levels. The band denoted with black arrows is total IRF3, coinciding with the 50-kilodalton protein marker. Protein markers are expressed in kilodaltons.

To determine if the cytoplasmic DNA and cGAS aggregates could activate innate signaling, we first determined if IRF3 was activated. When we measured IRF3 phosphorylation at serine residue 386 (p-IRF3 Ser386), a marker for IRF3 activation, at 1 to 6 h after *d*109 infection, we observed that IRF3 phosphorylation started from 2 hpi and increased significantly by 3 hpi in HSV-1 *d*109–infected HFF cells (Fig. 7*A*). Consistent with previous results in HFF cells (9), HSV-1 *d*109 infection triggered activation of IRF3 at 3 hpi requiring the cGAS/STING pathway, as evidenced by cGAS and STING protein depletions by siRNA-mediated knockdown and CRISPR Cas9-mediated knockout, respectively (Fig. 7*B*). We further confirmed that cGAS depletion did not intrinsically affect IRF3 activation, by showing that TLR4-dependent activation of IRF3 activation by lipopolysaccharide (LPS) was independent of cGAS (Fig. 7*C*). Thus, recognition of the nuclear DNA blebs by cGAS in the cytoplasm of HSV-1 *d*109–infected HFF cells is likely the initial event leading to the induction of IRF3.

Binding of HSV-1 *d*109 to cells was required for IRF3 activation as heparin treatment blocked IRF3 activation (Fig. 7*D*), and entry was required because anti-gD Mab blocked IRF3 activation (Fig. 7*E*). Similar to blebbing, nuclear transport of entering capsids was not needed for IRF3 activation (Fig. 7*F*). Thus, these results were consistent with *d*109 entry causing nuclear DNA blebbing, and cGAS sensing of the cytoplasmic DNA triggering IRF3 activation.

### Mechanism of Signal Inducing Blebbing: Role of Actin Contractility.

To examine the mechanism of the signaling following HSV-1 entry, we tested various inhibitors of cell signaling pathways. Inhibition of Ca^++^ signaling, PI3 kinase, IFI16, apoptosis, or endonucleases did not affect IRF3 signaling. However, inhibition of actin contractility did affect blebbing and IFN signaling. In those experiments, we either depolymerized or stabilized the filamentous actin cytoskeleton using latrunculin B (LB) or jasplakinolide (Jasp), or blocked the contractile motor protein, nonmuscle myosin II using blebbistatin (Bleb), as well as the master regulator of actin contractility, ROCK, using the Y-27632 inhibitor. IRF3 phosphorylation in HSV-1 *d*109–infected cells at 3 hpi was reduced in the presence of latrunculin B (LB), jasplakinolide (Jasp), and blebbistatin (Bleb) but not by Y-27632 (Fig. 7*G*). All three drugs reduced cytoplasmic cGAS foci (Fig. 7*J*) while LB and Jasp reduced extranuclear DNA (Fig. 7 *J* and *K*). LB also reduced ICP0 expression (Fig. 7*H*), so it could also affect entry. These results, in particular with Jasp which only partially reduced ICP0 expression but prevented cytoplasmic DNA and cGAS accumulation, argued that actin contractility could be involved in nuclear DNA blebbing and the downstream activation of the cGAS-STING pathway. Therefore, the myosin-actin contractility may have a role in cGAS sensing of cytoplasmic DNA whereas filamentous actin plays a role in transduction of the signal from to the nucleus to initiate blebbing.

### HSV-1 *d*109 Infection Elicits Interferon Protective Against HSV-1 Gene Expression in Secondary Cells.

To determine if the IRF3 activation by *d*109 resulted in biologically significant levels of type I interferon, we first tested the supernatant harvested from HSV-1 *d*109–infected HFF cells at 24 hpi for its ability to elicit STAT1 phosphorylation at tyrosine 701 (p-STAT1 Tyr701) in new cells, and we observed comparable levels of STAT1 phosphorylation by *d*109 infection culture supernatant to that induced by different doses of recombinant purified human IFNβ in the range of 50 to 1,000 IU/mL (Fig. 7*L*). In contrast, supernatant collected from HSV-1 *d*109–infected HFF cells at 1 hpi, after thorough rinsing of the infected cell monolayers, or those from mock-infected HFF cells collected at 1 and 24 hpi were unable to trigger STAT1 activation, arguing that the effect is attributed to the secretion of interferon by HSV-1 *d*109–infected HFF cells throughout the infection (Fig. 7*L*). Furthermore, 20 h-treatment with HSV-1 *d*109–infected cell supernatant collected at 24 hpi reduced expression of HSV-1 immediate ICP0 and ICP27 proteins at 6 hpi by replication-competent ICP0+ or ICP0- HSV-1 viruses (Fig. 7*M*). Remarkably, the ISG expression and antiviral effect achieved using supernatants were comparable to those observed after incubation of HFF with 100 IU/mL IFNβ (Fig. 7*M*). This argued that type I interferon in HSV-1 *d*109–infected cell supernatant induced by cGAS-STING-dependent IRF3 activation can exert anti-HSV-1 activity.

## Discussion

In this study, we discovered that infection with any of four viruses that enter normal human fibroblasts by fusion at the plasma membrane, HSV-1, HSV-2, HCMV, or HPIV3, induces blebbing of nuclear DNA at very early times of infection, resulting in cytoplasmic DNA, some of which colocalizes with the DNA sensor cGAS and activates IRF3 in a cGAS- and STING-dependent mechanism. HSV-1 activation of IRF3 leads to release of biologically active type I interferon from the cells. Use of the very sensitive PicoGreen dsDNA dye as a cytological stain (23) allowed us to discover these tiny cytoplasmic DNA structures. The rapid expulsion of nuclear DNA to the cytoplasm represents a new host response to viral infection in human fibroblasts and epithelial cells. As discussed below, there is prior evidence of virus- and bacterial-promoted fusion leading to innate signaling through cGAS-STING, but there was no previous evidence of nuclear DNA blebbing or cytoplasmic DNA. We will further examine each step of the process that we have observed.

### Fusion of the Viral Envelope with the Plasma Membrane and Viral Entry.

We observed that binding and entry of HSV-1 was required for DNA blebbing and did not require transport of nucleocapsids to the nuclear pore or viral gene expression. This was consistent with earlier reports that innate signaling can partially occur early after HSV-1 infection (11, 12), and even in the absence of viral gene expression (8, 10).

The ability of HSV, HCMV, and HPIV3 to fuse their envelopes with the host cell plasma membrane for virus entry and to induce nuclear DNA blebbing and the inability of VSV, which enters through a pH-dependent endosomal pathway (28) to induce DNA blebbing argues that the effect is due to fusion of the viral envelopes at the plasma membrane. Analysis of additional viruses and viral fusogens, potentially with viral pseudotypes, will further test the hypothesis that the nuclear DNA blebbing is due to viral fusion.

Previous studies have reported that viral or bacterial infection can induce cGAS-STING signaling pathways (29–31). SARS-CoV-2 spike protein-induced cell fusion (32, 33), and the syncytia formed by a *Burkholderia* bacterium-encoded T6SS5 fusogen (34) result in the formation of cytoplasmic micronuclei that are sensed by cGAS. Also, the cellular fusion-from-within upon the overexpression of the reptilian reovirus p14 protein results in STING-dependent IRF3 activation (35). However, the mechanism of these effects was not defined. There was no previous observation of nuclear DNA blebbing under these conditions. Induction of cell–cell fusion by PEG did not induce nuclear DNA blebbing, which argues that virus envelope fusogen proteins are required for the effect.

### Transmission of the Signal to the Nucleus.

We tested multiple cellular signaling pathways, but intact actin microfilaments and their contractility are thus far uniquely required for this nuclear DNA blebbing and activation of IRF3-dependent innate immune responses. We hypothesize that the actin cytoskeleton mechanically transduces the fusion of the viral envelope with the plasma membrane by a contractility mechanism causing breakage of the nuclear membrane and releasing nuclear DNA into the cytoplasm, thus impacting the nuclear envelope integrity. This is supported by the fact that the plasma membrane and nuclear envelope are both scaffolded by the actin cytoskeleton (reviewed by refs. 36 and 37). Activation of actin contractility may occur as a result of changes in the membrane curvature due to multiple fusion events induced by the multivalent virion particles. Actin contractility has been reported previously to cause nuclear blebbing and rupture (38) which is consistent with our observations. Similar events have also been documented for other environmental mechanical stresses (38) like confined cell migration (39). It has been reported that HSV activates actin modulatory and mechanotransduction factors very early in infection, including Rac1, Cdc42 (40, 41), focal adhesion kinase (FAK) (42), or inositol triphosphate receptor (IP3R)-dependent activation of calcium pathways (43). In Vero cells, HSV-1 enters through fusion at the plasma membrane, and the actin-depolymerizing drug cytochalasin D does not influence virus entry, suggesting that intact actin microfilaments are not required for viral envelope fusion with the plasma membrane (26). Thus, the mechanosensing system encompassing the cortical actin (reviewed in ref. 44), the actin regulatory factors (45), and the actin-interacting proteins at the outer surface of the nuclear envelope (36) need to be thoroughly investigated to further decipher the exact determinants of the virus-induced nuclear DNA blebbing.

### Blebbing of DNA from the Infected Cell Nucleus and Colocalization with cGAS.

This rapid response targeting the host cell nucleus diverges from other types of nuclear DNA leakage into the cytoplasm, like micronuclei, which usually involve cell division aberrancies (reviewed in refs. 46 and 47) and independent of newly synthesized viral gene products. Most of the cytoplasmic blebs that we observed appear to be free of nuclear envelope or nuclear lamina, making them different from micronuclei. TEM analysis of the nuclear envelope of HSV-infected cells showed areas of separation between the inner and outer nuclear membranes, and serial sectioning revealed disruptions in both membranes. Immunogold labeling of DNA showed nuclear DNA streaming through disruptions, indicating that DNA expulsion occurred without nuclear membranes surrounding them. These TEM findings confirm the observations made by light microscopy.

The expelled nuclear DNA is at least in part accessible to cGAS, which forms aggregates around it and induces the IRF3-dependent type I interferon production through the cGAS-STING pathway. Our results also confirm the cGAS protein as the chief host factor in the type I interferon production in response to HSV-1 entry in HFF cells, considering that its ligand in HSV-1-infected HFF cells was unknown. In the HFF model, the fact that the cytoplasmic PicoGreen stain does not label entering capsids is indicative of a nuclear DNA blebbing event.

Several ligands have been proposed for the interaction of HSV with the cGAS-STING pathway, but the ligand for cGAS in HSV-infected cells has remained elusive. First, it has been assumed since the discovery of cGAS that its ligand in HSV-infected cells is HSV DNA, leaking either from capsids or the nucleus (14). Direct measurements of viral DNA in cGAS pulldowns found only 0.2% of the DNA sequences associated with cGAS are HSV-1 DNA in WT infected cells at 4 hpi, and the majority of associated sequences was not identified (48). Second, the HSV-1 UL12.5 late gene product promotes mitochondrial DNA degradation (49), which could provide cytoplasmic DNA for activation of cGAS. However, UL12.5 is expressed as a late protein in WT virus–infected cells (7) and not in *d*109 virus–infected cells, so mitochondrial DNA degradation does not explain our results in *d*109 and early WT virus infection. Third, degradation of HSV-1 capsids in macrophages can release viral DNA into the cytoplasm for recognition by DNA sensors (50), but this is a nonproductive infection situation unlike that in fibroblasts and keratinocytes. Fourth, a recent paper showed that the HSV-1 ICP0 protein provokes centromeric DNA amplification and the activation of nuclear cGAS (18) in dendritic cells at 24 hpi. The phenotype presented here is ICP0-independent and shows cytoplasmic cGAS coating cytoplasmic DNA, so it is a new mode of sensing HSV by cGAS.

### Comparison of Mechanisms of DNA Sensing and Innate Signaling.

Several mechanisms are likely to detect foreign DNA during viral infection, and it is useful to consider these are early and late events. We observed nuclear DNA blebbing that is triggered upon a very early event, 1 to 3 hpi, in fibroblasts and keratinocytes infected with nuclear DNA viruses and a cytoplasmic RNA virus, human parainfluenza virus. With HSV, this does not require any de novo viral gene expression. In macrophages, which are nonpermissive for HSV, incoming capsids are degraded in the cytoplasm and incoming virion DNA is recognized by IFI16 by 4 hpi (50). At late times postinfection, several viruses including dengue virus (51) and even HSV-1 (49) can induce release of mitochondrial DNA into the cytoplasm. Replicated viral DNA may even leak into the cytoplasm for recognition by cGAS, although the bulk of progeny viral DNA is kept within replication compartments and protected from DNA sensors.

### Limitations of this Study.

HSV-1 infection activates IFN signaling via cytosolic (cGAS and RIG-I), and endosomal (TLR3 and TLR9) sensors, but counters this through HSV-1 gene products including ICP0, US3, US11, and UL41/vhs to inhibit IFN signaling (7). Thus, it is difficult to parse out the importance of this one new mechanism of IFN induction in overall pathogenesis of HSV. Furthermore, innate signaling mechanisms often involve host pathways that serve other biological functions, so it is unlikely that there is a dedicated host mechanism for this new pathway that can be inactivated to allow a clear parsing out of its overall significance. Notably, this HSV-1 entry-induced nuclear DNA blebbing and cGAS activation occur within 1 hpi, before the viral IFN antagonists can intervene, revealing a novel fusogen-driven mechanosensing pathway that precedes canonical immune sensor activation. Viral entry by these same viruses by other mechanisms, such as viral spread through intercellular connections, may evade this host response.

In conclusion, the expulsion of nuclear DNA into the cytoplasm of virus-infected cells upon virus-cell plasma membrane fusion represents a novel and immediate cellular innate response, mounted through the cGAS-STING-IRF3-type I IFN signaling node, that seems likely to play a role in successfully preventing the spread of viruses to surrounding uninfected cells.

## Materials and Methods

Detailed materials and methods can be found in the *SI Appendix*.

### Cell Culture.

Primary human foreskin fibroblasts (HFF cells) (ATCC; CRL-1635) were cultured as described previously (5) in Dulbecco’s modified Eagle medium (DMEM; Corning; 10-017-CV) containing 10% volume/volume (v/v) heat-inactivated fetal bovine serum (FBS; Gibco) and 1% (v/v) penicillin-streptomycin (Pen-Strep; Gibco; 15140-122; 10,000 units/mL of penicillin and 10,000 μg/mL of streptomycin). The human normal oral gingival keratinocytes (NOK cells) were immortalized through the human telomerase reverse transcriptase (hTERT) (52) and were a kind gift from K. Munger (Tufts University School of Medicine, Boston, MA). NOK cells were maintained in keratinocyte serum-free medium (Gibco; 17005042) supplemented with 1% (v/v) penicillin-streptomycin. The African green monkey kidney Vero cells (ATCC; CCL-81) were cultured in DMEM supplemented with 5% (v/v) heat-inactivated bovine calf serum (BCS; Gibco), 5% (v/v) FBS, and 1% (v/v) penicillin-streptomycin. The Vero-derived FO6 complementing cell line expressing HSV-1 ICP0, ICP4, and ICP27 proteins (53) was generously provided by N. DeLuca (University of Pittsburgh, Pittsburgh, PA). FO6 cells were maintained in DMEM containing 10% (v/v) FBS, and 300 μg/mL hygromycin B (Gibco/Life Technologies; 10687-010) and 500 μg/mL geneticin (G418; Life Technologies; 11811031). CRISPR Cas9-based knockout cells were isolated as described previously (54). These two cell lines were cultured in HFF medium supplemented with 1 μg/mL puromycin.

### Viruses.

HSV-1 wild-type KOS (55, 56) was propagated and titrated using plaque assays on Vero cells as described (57). N. DeLuca kindly provided the HSV-1 *d*109 mutant strain (24). The HSV-1 ICP0 null mutant virus (HSV-1 7134) and HSV-1 ICP0 rescued virus (HSV-1 7134R) (58) were propagated and titrated by plaque assay in U2OS cells. The HSV-1 *d*109 strain was propagated and titrated by plaque assay on the FO6 complementing cell line. HSV-2 wild-type G (59) was propagated and titrated by plaque assay on Vero cells. The HSV-1 KOS *d*38 virus, which contains a deletion in the *UL38* gene, was constructed, propagated, and tittered in V38 cells, which contain the HSV-2 *UL38* gene and provided by Changhong Zhou. The human cytomegalovirus (HCMV) AD169 strain (60), a generous gift from T. Kowalik (University of Massachusetts, Worcester, MA), was titrated on HFF cells. The human parainfluenza virus 3 (HPIV3) used is a clinical isolate encoding an F protein harboring a lysine in residue 108 (F K108) and an eGFP cassette (HPIV3 F K108 eGFP) (61). The HPIV3 stock was propagated on human lung adenocarcinoma Calu-3 cells and titrated on Vero cells. The recombinant vesicular stomatitis virus strains expressing the firefly luciferase or enhanced GFP were generated and rescued from cDNA as previously reported (62–64). The rVSV-Luc and rVSV-GFP strains were kindly provided by A. Lee (Dana-Farber Cancer Institute, Boston, MA) and S. Whelan (Harvard Medical School, Boston, MA), respectively. rVSV stocks were propagated on the hamster kidney BHK-21 cell line and titrated on Vero E6 cells using plaque assay. The wild-type herpesviruses and HPIV3 stocks used in this study tested negative for *Mycoplasma* contamination using a PCR-based *Mycoplasma* detection kit (Venor™ GeM *Mycoplasma* Detection Kit; Sigma; Mp0025-1kt), ruling out bacterial contamination as the source of PicoGreen-labeled cytoplasmic DNA structures.

### Virus Infections.

Virus infections were performed as reported (8). Complete protocols for virus infections are reported in the *SI Appendix*.

### Bioactive Reagents.

Information about lipopolysaccharides, human recombinant IFNβ and IFN bioassays, virus entry inhibitors, and drugs targeting actin cytoskeleton can be found in the *SI Appendix*.

### PicoGreen Staining.

Staining of total DNA with PicoGreen using supernatant virus stocks (65) were used for infections in these experiments to minimize the DNA and debris is described in the *SI Appendix*.

### Immunofluorescence.

Immunofluorescence experiments were conducted using the procedure described before (66), with slight adaptations. Complete protocols for immunofluorescence assay, 3-D rendering of confocal micrographs, orthogonal views and Z-projection generation, and colocalization studies are reported in the *SI Appendix*.

### Visualization of Input EdC-Labeled Viral Genomes Using Click Chemistry.

The 5-ethynyl-2′-deoxycytidine (EdC)-labeled HSV-1 WT KOS virus stocks were generated and click chemistry reactions were performed as reported before (54). In-depth description of the procedures can be found in the *SI Appendix*.

### Western Blotting Assays.

Western blotting assays were carried out as in ref. 67. Full protocol and antibodies used can be found in the *SI Appendix*.

### Knockdown Studies.

siRNA-based knockdown studies were conducted as previously described (67), with minor modifications for optimal cGAS knockdown. Detailed information about the procedure is found in the *SI Appendix*.

### Plasmid DNA Transfection.

Plasmid DNA transfections were performed as (9). Details of plasmid DNA transfection are described in the *SI Appendix*.

### Virus Neutralization.

Procedures for HSV-1 neutralization with DL11 anti-gD antibody are reported in the *SI Appendix*.

### HFF Cell Fusion with PEG.

The polyethylene glycol-driven cell fusion protocol was modified slightly from that previously reported (68). The complete procedure is reported in the *SI Appendix*.

### Transmission Electron Microscopy (TEM) Analyses and Immunogold Labeling of DNA.

Detailed protocols for electron microscopy analyses are provided in the *SI Appendix*.

### Graphs and Statistical Analyses.

Data were graphed using GraphPad Prism software version 10.2.0. The one-tailed paired Student’s *t* tests were launched in the Microsoft Excel software version 16.89.1, with statistical significance with a *P*-value lower than or equal to 0.05.

## Supplementary Material

Appendix 01 (PDF)

## Data Availability

Study data are included in the article and/or *SI Appendix*.
