# Supplementary material for: Expulsion of nuclear DNA to the cytoplasm after viral entry: A mechanism for activation of the cGAS pathway
Source: Proc Natl Acad Sci U S A. 2026 Aug 18;123(34):e2614472123. doi: 10.1073/pnas.2614472123 (PMC13506004; doi:10.1073/pnas.2614472123)
Supplement: Supplementary file 1 [file pnas.2614472123.sapp.pdf]

PNAS

Supporting Information for

Expulsion of Nuclear DNA to the Cytoplasm after Viral Entry: A Mechanism for Activation of the cGAS Pathway

Nicolás Romero, Hyung Suk Oh, Max E. Mertens, Maria Ericsson, Kyle N. Stearns, Anne Moscona, David M. Knipe.

David M. Knipe

Email: david_knipe@hms.harvard .edu

Supporting Information Text

Detailed materials and methods

Cell culture. Primary human foreskin fibroblasts (HFF cells) (ATCC; CRL-1635) were cultured as described previously (1) in Dulbecco's modified Eagle medium (DMEM; Corning; 10-017-CV) containing 10% volume/volume (v/v) heat-inactivated fetal bovine serum (FBS; Gibco) and 1% (v/v) penicillin-streptomycin (Pen-Strep; Gibco; 15140-122; 10,000 units/ml of penicillin and 10,000 µg/ml of streptomycin). The human normal oral gingival keratinocytes (NOK cells) were immortalized through the human telomerase reverse transcriptase (hTERT) (2), and were a kind gift from K. Munger (Tufts University School of Medicine, Boston, MA, USA). NOK cells were maintained in keratinocyte serum-free medium (Gibco; 17005042) supplemented with 1% (v/v) penicillin-streptomycin. The African green monkey kidney Vero cells (ATCC; CCL-81) were cultured in DMEM supplemented with 5% (v/v) heat-inactivated bovine calf serum (BCS; Gibco), 5% (v/v) FBS, and 1% (v/v) penicillin-streptomycin. The Vero-derived FO6 complementing cell line expressing HSV-1 ICP0, ICP4, and ICP27 proteins (3) was generously provided by N. DeLuca (University of Pittsburgh, Pittsburgh, PA, USA). FO6 cells were maintained in DMEM containing 10% (v/v) FBS, and 300 µg/ml hygromycin B (Gibco/Life Technologies; 10687-010) and 500 µg/ml geneticin (G418; Life Technologies; 11811031).

CRISPR Cas9 control and STING knockout HFF cells. CRISPR Cas9-based knockout cells were isolated as described previously (4). These two cell lines were cultured in HFF medium supplemented with 1 µg/ml puromycin.

Viruses. HSV-1 wild-type KOS (5, 6) was propagated and titrated using plaque assays on Vero cells as described (7). The HSV-1 ICP0null (HSV-1 7134) and HSV-1 ICP0null rescue (HSV-1 7134R) (8) were propagated and titrated by plaque assay in U2OS cells. N. DeLuca kindly provided the HSV-1 *d*109 mutant strain (9). The HSV-1 *d*109 strain was propagated and titrated by plaque assay on the FO6 complementing cell line. HSV-2 wild-type G (10) was propagated and titrated by plaque assay on Vero cells. The HSV-1 KOS d38 virus, which contains a deletion

in the *UL38* gene, was constructed, propagated, and tittered in V38 cells, which contain the HSV-2 *UL38* gene (11). The human cytomegalovirus (HCMV) AD169 strain (12), a generous gift from T. Kowalik (University of Massachusetts, Worcester, MA, USA), was titrated on HFF cells. The human parainfluenza virus 3 (HPIV3) used is a clinical isolate encoding an F protein harboring a lysine in residue 108 (F K108) and an eGFP cassette (HPIV3 F K108 eGFP) (13). The HPIV3 stock was propagated on human lung adenocarcinoma Calu-3 cells and titrated on Vero cells. The recombinant vesicular stomatitis virus strains expressing the firefly luciferase or enhanced GFP were generated and rescued from cDNA as previously reported (14-16). The rVSV-Luc and rVSV-GFP strains were kindly provided by A. Lee (Dana-Farber Cancer Institute, Boston, MA, USA) and S. Whelan (Harvard Medical School, Boston, MA, USA), respectively. rVSV stocks were propagated on the hamster kidney BHK-21 cell line and titrated on Vero E6 cells using plaque assay. The wild-type herpesviruses and HPIV3 stocks used in this study tested negative for mycoplasma contamination using a PCR-based *Mycoplasma* detection kit (Venor™ GeM Mycoplasma Detection Kit; Sigma; Mp0025-1kt), ruling out bacterial contamination as the source of PicoGreen-labeled cytoplasmic DNA structures.

Virus infections. Virus infections were performed as reported (17). In brief, virus inoculum was diluted in sterile phosphate-buffered saline (PBS; Boston BioProducts; BM-220) supplemented with 1% (v/v) heat-inactivated BCS and 0.1% weight/volume (w/v) glucose (PBS-glu-ICS) and incubated for 1 h, at 37ºC in a rocking incubator at 50 revolutions per min (rpm). Subsequently, the inoculum was removed, and the infected cell monolayers were overlaid with fresh cell culture medium and incubated at 37ºC and 5% CO_2 until the indicated harvest time. The figure legends indicate whether virus adsorption was performed in the presence or absence of a specific inhibitor or HSV-1 neutralizing antibody. The multiplicity of infection (MOI) is specified in each figure legend and it is measured as plaque-forming units per cell (pfu/cell).

rVSV-Luc and rVSV-GFP infections were synchronized (Supp. Fig. 1E & 1H). To this end, HFF cells were pre-chilled at 4ºC for 30 min before being incubated with ice-cold virus inoculum. Virus adsorption was conducted at 4ºC for 1 h, followed by a switch to 37ºC and 5% CO_2, removal of

the inoculum at 1 hpi, overlaying the cell culture with HFF medium, and then incubation at 37ºC and 5% CO_2. For virus inactivation at the cell surface, treatment with citric acid buffer (40 mM citric acid, 10 mM KCl, 150 mM NaCl, pH 3.5) was conducted at room temperature for 1 min, and two washing steps with PBS-glu-ICS were done prior to overlaying cells with medium.

Bioactive reagents. Human recombinant IFNβ obtained from R&D Systems (8499-IF) was reconstituted in ultrapure sterile water. Human recombinant IFNβ was utilized in concentrations from 10 to 1000 IU/ml, as described in the legend of Fig. 7. Heparin (Sigma, H3149) stock was made in PBS-glu-ICS, whereas colchicine (Sigma, C3915) and chloroquine (Sigma, C6628) stocks were made in sterile PBS. Heparin was used at 50 μg/ml, colchicine at 5 μM, and chloroquine at 100 μM.

The G-actin stabilizer latrunculin B from *L. magnifica* (Sigma, 428020), the F-actin stabilizer jasplakinolide from *J. johnstoni* (Sigma, 420127), the inhibitor of the non-muscle myosin II (NMII) blebbistatin (Selleckchem, E1249), and the ROCK inhibitor Y-27632 (Y-27632 2 HCl, DNSK International, DNSK-KI-15-02) were resuspended in sterile dimethyl sulfoxide (DMSO, Sigma, D5879). Latrunculin B, blebbistatin, and Y-27632 were used at 10 μM, whilst jasplakinolide was utilized at 200 nM. An equivalent concentration of DMSO was used as vehicle control for treatments (0.1% v/v). Lipopolysaccharides (LPS) from *E. Coli* O111:B4 were obtained from Sigma (L4391), resuspended in ultrapure sterile water, and used in cell cultures at 1 μg/ml.

PicoGreen staining. Cells were seeded on sterile coverslips (FisherBrand). Supernatant virus stocks (18) were used for infections in these experiments to minimize the DNA and debris added to cells. At the indicated time point, cells were rinsed three times with PBS and fixed with 3.7% formaldehyde (Sigma, F8875) in PBS for 10 min at room temperature. After three PBS washes, cell membranes were stained with 5 μg/ml Texas red-conjugated wheat germ agglutinin (Tx-Red WGA; Invitrogen; W21405) for 10 min at room temperature in the dark. Later, unbound Tx-Red WGA was removed by three washes with PBS, and then, cells were permeabilized with 0.1% (v/v) Triton X-100 solution (Sigma, T8787) for 10 min. Cells were incubated with PicoGreen stock

(Quant-iT™ PicoGreen™ dsDNA reagent; Invitrogen; P7581) diluted 1:1000 in PBS for 1 h at 37ºC. Upon the removal of the unbound PicoGreen with 3 PBS washes, coverslips were mounted on 3 µl-drops of mounting medium (SlowFade™ Diamond Antifade Mountant; Invitrogen) on microscopy slides (VWR North American) and sealed with clear nail polish. Imaging was performed using a Nikon Ti inverted confocal microscope with a W1 Yokogawa Spinning disk (50 µm pinhole disk), with an Andor Zyla 4.2 Plus sCMOS monochrome camera through the Nikon Elements Acquisition Software AR 5.02 and a 100x objective lens Plan Apo λD 100x/1.45 Oil DIC using immersion oil type 37 (Cargille laboratories) at the Microscopy Resources on the North Quad (MicRoN) imaging facility at Harvard Medical School. Images were analyzed using ImageJ Version 2.14.0 (NIH). In the quantitation of extranuclear PicoGreen-stained structures, either for the calculation of the percentage of positive cells for extranuclear DNA, or number of structures per cell, the orthogonal view tool was used to analyze Y/Z axes (Image>Stacks>Orthogonal Views) after merging channels corresponding to PicoGreen and Tx-Red WGA (Image>Color>Make Composite). These analyses were done on 3-4 100x magnification fields per condition/experiment, captured through 200 nm-width Z-stacks comprising the entire cell volume in the Z axis. Cytoplasmic PicoGreen-labeled dots were considered only when within the cell boundaries defined by the Tx-Red WGA fluorescent signal.

Immunofluorescence. Immunofluorescence experiments were conducted using the procedure described before (19), with slight adaptations. The primary antibodies and their working dilutions were the following: anti-ICP5 antibody (1:500 dilution, East Coast Bio/Abnova, HA018, MAB13576), anti-cGAS antibody (1:100 dilution, Cell Signaling Technology, 79978), anti-lamin A/C antibody (1:100 dilution, Cell Signaling Technology, 4777), anti-beta-I/II-tubulin antibody (1:200 dilution, Sigma, T8535), anti-IFI16 (1:50 dilution, Sigma, IFI-230), and anti-lamin B1 (1:500 dilution, Abcam, ab16048). The next day, after 3 PBS washes a 1:500 dilution of the corresponding fluorochrome-labeled goat-raised secondary antibodies (from Invitrogen: A11001, A11032, A11072, A21236, and A21246; and from Abcam: ab150115) diluted in 5% NGS solution for 1 h at 37ºC. Upon removing the unbound secondary antibody, PicoGreen (1:1000 dilution)

was added for 1 h at 37ºC. After three washing steps with fPBS, coverslips were mounted and sealed as described in the *PicoGreen staining* section. The imaging and image analyses were also performed as above. As earlier indicated, virus supernatant stocks were used to minimize cell debris.

Fluorescence microscopy was conducted for the detection of rVSV-GFP infection (Supp. Fig. 1H). Mock-infected and virus-infected cell nuclei were counterstained using a 1 μg/ml DAPI solution (ThermoFisher) after Triton X-100-based cell membrane permeabilization, as indicated above. Fixation, permeabilization, coverslip mounting, and image taking were identical to those described for immunofluorescence assays. For this specific experiment, images were captured using a Plan Apo λD 60x/1.42 Oil DIC objective lens with immersion oil type 37.

Z-projections were generated using confocal microscopy images captured with 200 nm-width Z-stacks spanning the full cell volume in the z axis in the ImageJ image analysis software (Image>Stacks>Z-projection>Max intensity).

Three-dimensional rendering using Arivis software. Raw images as nd2 files were converted into sis files using Arivis SIS Converter Version 3.4.0 to be opened with Arivis Vision 4D Version 4.1.2 image analysis software (Zeiss), provided by MicRoN imaging facility at Harvard Medical School. The confocal microscopy images, taken with Z-stack width of 200nm, were visualized in a 4D viewer mode (View>Show as 4D viewer>Volumetric visualization). 3-D images were rotated and zoomed to visualize the cytoplasmic DNA structures and cGAS and lamin A/C proteins from the cell apical view. The modeled DNA surfaces based on PicoGreen fluorescent signal, illustrated in the bottom grey-scale panel of Fig. 1D, were generated by the tool "Selecting a specific area of the model in a 3D environment" by clicking on a determined cell upon volumetric visualization.

Co-localization studies. The Pearson's correlation coefficient R(obs) was calculated using the co-localization test tool (Analyze>Colocalization test) from ImageJ software on split channels, either corresponding to cGAS or HSV-1 ICP5 stains, by defining 3 x 3 μm squares in the cell

cytoplasm (Edit>Selection>Specify>Constrain square/circle). The pixel size was 130 nm, and the Costes method was applied.

Visualization of input EdC-labeled viral genomes using click chemistry. The 5-ethynyl-2'-deoxycytidine (EdC)-labeled HSV-1 WT KOS virus stocks were generated as reported before (4). Infections with the EdC-labeled HSV-1 wild-type KOS were conducted identically as with EdC-free viruses using coverslips. Cell monolayers were rinsed three times with sPBS before fixation at the indicated time point with a 3.7% formaldehyde solution for 10 min at room temperature. Cell permeabilization, blocking, and incubation with primary antibodies, the anti-lamin B1 and anti-ICP5 antibodies, and fluorochrome-conjugated secondary antibody were carried out as described in the *Immunofluorescence assay* section. Next, cells were incubated with the click chemistry reaction solution as done previously (4).

MitoTracker staining. Live HFF cells, infected or not with HSV-1 *d*109, were incubated at 2.5 hpi for 30 min at 37ºC and 5% CO_2 with 500 nM MitoTracker Orange CMTMRos (Invitrogen, M7510) prior to fixation at 3 hpi. MitoTracker probe was washed three times with PBS before adding the fixative. Fixation, permeabilization and PicoGreen staining were conducted as in *PicoGreen staining* section. MitoTracker probe was a kind gift from D. Sinclair laboratory (Harvard Medical School, Boston, MA, USA).

Western blotting assays. Western blotting assays were carried out as in (20). The primary antibodies used for Western blotting were anti-GAPDH antibody (1:20000 dilution, Abcam, ab8245), anti-ICP0 antibody (1:2000 dilution, East Coast Bio, H1A027), anti-ICP27 antibody (1:2000 dilution, East Coast Bio, P1119), anti-cGAS antibody (1:1000 dilution, Cell Signaling Technology, 79978), anti-p-IRF3 Ser386 (1:1000 dilution, Abcam, ab76493), anti-IRF3 antibody (1:1000 dilution, Cell Signaling Technology, 4302), anti-STING antibody (1:1000 dilution, Cell Signaling Technology, 13647), anti-p-STAT1 Tyr701 antibody (1:1000 dilution, Cell Signaling Technology, 9167), anti-STAT1 antibody (1:1000 dilution, Cell Signaling Technology, 9176), anti-

MxB antibody (1:1000 dilution, Novus Biologicals, NBP1-81018) and anti-IFI16 antibody (1:1000 dilution, Abcam, ab55328). The day after, the unbound primary antibody was removed with serial washing steps with PBS with 0.1% (v/v) Tween 20 (namely PBS-T) before adding the goat-raised horse radish peroxidase (HRP)-conjugated secondary antibodies, either goat anti-mouse IgG HRP (1:2000 dilution, Cell Signaling Technology, 7076) or goat anti-rabbit IgG HRP (1:2000 dilution, Cell Signaling Technology, 7074) diluted in blocking buffer. Blots were incubated for 1 h at room temperature with the HRP-conjugated secondary antibodies. Upon washing the unbound secondary antibody with PBS-T, blots were incubated with chemiluminescence reagents, including Immobilon Classico Western HRP substrate (Millipore, WBLUC0500), Immobilon Forte Western HRP substrate (Millipore, WBLUF0500), and SuperSignal™ West Femto Maximum Sensitivity Substrate (ThermoFisher), depending on the target. Blots were exposed to autoradiography films (HyBlot CL® Autoradiography Film, Thomas Scientific) and developed using an M35A X-Omat processor (Kodak). Autoradiography films were scanned using a CanoScan 8800F scanner (Canon) and VueScan 9 x64 software. Protein markers (Precision Plus Protein Dual Color Standards, BioRad, 1610374) are expressed in kilodaltons in each Western blot experiment.

Knockdown studies. siRNA-based knockdown studies were conducted as previously described (20), with minor modifications for optimal cGAS knockdown. HFF cells, 10^5 /well, were seeded on MW12 plates (Corning). The day after, annealed non-target siRNAs (ON-TARGETplus Non-targeting Control Pool, Dharmacon) or cGAS siRNAs (ON-TARGETplus Human MB21D1 siRNA, Dharmacon), re-suspended in nuclease-free water, were diluted in 100 µl of OPTI-MEM (Opti-MEM™ I Reduced Serum Medium, Gibco,) for a final concentration of 2 µM. In parallel, 6 µl of RNAiMAX lipofectamine (Lipofectamine™ RNAiMAX Transfection Reagent, Invitrogen) were diluted in 100 µl of OPTI-MEM. Lipofectamine was dropwise mixed with the siRNA solution, and after gentle vortexing, siRNA/lipofectamine complexes were allowed to form for 5 min at room temperature. Then, the siRNA/lipofectamine complexes were added directly to the HFF cells,

diluting them in the cell culture medium, at a final concentration of 20 nM. At 48 h post transfection, siRNA-transfected cells were subject to infection or treatment.

Plasmid DNA transfection. Plasmid DNA transfections were performed as (21). The pUC19 plasmid (New England Biolabs; N3041A) was propagated on and isolated from DH5α *E. Coli* competent cells (New England Biolabs) using the QIAprep Spin Miniprep Kit (Qiagen). Plasmid DNA concentration was determined using the Nanodrop Lite spectrophotometer (ThermoFisher). Every plasmid DNA transfection in the present study was done using 200 ng of pUC19 plasmid, which was diluted in 100 µl of OPTI-MEM (Gibco) with 0.5 µl of PLUS reagent (PLUS™ Reagent, Invitrogen; 10964-021). Concomitantly, 2.5 µl of LTX lipofectamine (Lipofectamine™ LTX Reagent, Invitrogen) was diluted in another tube in 100 µl of OPTI-MEM, and then lipofectamine was added to the plasmid dilution. Upon thoroughly vortexing the mixture, it was incubated at room temperature for 5 min. Next, the plasmid DNA-lipofectamine complexes were directly added to HFF cells.

Virus neutralization. The purified mouse monoclonal neutralizing anti-gD DL11 antibody was kindly provided by G. Cohen and R. Eisenberg (University of Pennsylvania, Philadelphia, PA). The DL11 antibody at a concentration of 5 µg/ml was mixed with 10^5 plaque-forming units (pfu) of supernatant virus stocks of HSV-1 wild-type or HSV-1 *d*109, diluted in sPBS in a final volume of 200 µl and incubated for 16 h at 4ºC for virus neutralization upon thorough vortexing. The next day, tubes were warmed at 37ºC and added to the cell cultures for 1 h at 37ºC, enabling virus adsorption. At 1 hpi, cell monolayers were rinsed three times with sPBS, and the HFF medium was overlaid onto cells before incubation at 37ºC and 5% CO_2. Virus neutralization efficacy was determined through the Western blotting-based detection of ICP0 protein accumulation at 6 hpi in the presence or absence of DL11 antibody in HSV-1 WT-infected HFF cells (Fig. 3F).

HFF cell fusion with PEG. The polyethylene glycol-driven cell fusion protocol was modified slightly from that previously reported (22). Confluent HFF cells were detached using a 1x 0.05%

trypsin-0.53mM EDTA solution (Corning) and washed in 30 ml of serum- and antibiotic-free DMEM. Pellets containing 2×10^6 HFF cells were either resuspended in 1 ml of DMEM or 50% (w/v) polyethylene glycol 3350 (PEG 3350; Sigma) diluted in DMEM and incubated for 2 min at room temperature. Then, the concentration of PEG 3350 was lowered to 5% (w/v) using DMEM, allowing the cells to fuse for an additional 30 min at room temperature. After two consecutive washing steps with 30 ml of DMEM, HFF cells were resuspended in complete HFF medium, seeded on coverslips in 24-well plates using 3×10^5 cells/well, and incubated for an additional 2.5 h at 37ºC and 5% CO_2. Then, cells were fixed and stained with PicoGreen and WGA as described in the *PicoGreen staining* section. Imaging was conducted as explained above. Polykaryons were considered as two or more PicoGreen-labeled cell nuclei within the same cytoplasmic contour defined by WGA membrane stain.

Interferon bioassays. The HFF cells were infected with HSV-1 *d*109 mutant, and at 1 hpi, the virus inoculum was washed away three consecutive times with sPBS. Then, the cell monolayer was overlaid with HFF medium. Cell supernatants were collected at 1 hpi, before cell monolayers are rinsed, or 24 hpi and immediately stored at -80ºC. For the interferon bioassays, supernatants were warmed at 37ºC. They were added, together with the stated units of recombinant purified human IFNβ diluted in pre-warmed HFF medium, to new HFF cells for 30 min at 37ºC and 5% CO_2 to evaluate STAT1 phosphorylation. Recombinant purified human IFNβ international units (IU) were determined in human cervix adenocarcinoma HeLa cells by the manufacturer (R&D Systems), where 1 µg of cytokine corresponds to 2.8 x 10^5 IU.

Transmission electron microscopy (TEM) analyses. HFF cells were seeded into 24-well plates on sterile Aclar plastic coverslips. The following day, cells were either infected with HSV-1 *d*109 supernatant virus stock or left uninfected as control. At 3 hpi, both infected and uninfected cell monolayers were rinsed with sPBS and fixed using a 1:1 mixture of 2x fixative solution and HFF cell culture medium. The resulting final fixative concentrations were 1.25% (v/v) paraformaldehyde, 2.5% (v/v) glutaraldehyde, and 0.03% (v/v) picric acid in 0.1 M sodium

cacodylate buffer (pH 7.4). Cells were fixed for at least 1 h at room temperature. Following fixation, cells were washed with 0.1 M sodium cacodylate buffer and incubated for 1 h in an aqueous solution containing 1% osmium tetroxide (OsO_4) and 1.5% potassium ferrocyanide ($KFeCN_6$). Samples were then washed twice with water and once with 50 mM maleate buffer (pH 5.5), followed by incubation in 1% uranyl acetate diluted in maleate buffer for 1 h. After staining, samples were washed once with maleate buffer and twice with water and then dehydrated through a graded ethanol series (50%, 70%, 95%, and 2x 100%) for 5 min at each step. Samples were subsequently embedded in TAAB Epon resin by first blotting off excess ethanol, followed by placing the Aclar coverslip (cell side down) onto a drop of TAAB epon on a clean Aclar disc. They were then polymerized at 60ºC for 48 h.

After polymerization, the Aclar discs were removed, and a 1 × 1 mm region containing flat-embedded cells was excised and mounted onto an Epon block for ultrathin sectioning. Ultrathin (80nm) sections were cut using a Reichert Ultracut-S microtome, collected on copper grids, and stained with 0.2% lead citrate.

Immunogold labeling of DNA. Immunogold labeling was performed using the Tokuyasu method (23). HFF cells were either infected with HSV-1 *d*109 lysate stock or left uninfected and harvested at 3 hpi. Cell monolayers were rinsed once with sPBS, and cells were detached by incubation with 1× 0.05% trypsin–0.53 mM EDTA solution (Corning) for 3-5 min at 37ºC in 5% CO_2. Cells were gently pelleted at 220 × g for 5 min at 4ºC, washed once with 1 mL sPBS, and pelleted again. Cell pellets were fixed by carefully overlaying a 4% formaldehyde solution in 0.1M PBS pH 7.4 and incubated for 2 h at room temperature without disturbing the pellet. Following fixation, pellets were infiltrated with a solution of 2.3 M sucrose and 0.2 M glycine in PBS for 15 min, and then rapidly frozen in liquid nitrogen.

Ultrathin cryosectioning was performed at −120ºC. Sections were collected on formvar-carbon–coated copper grids and floated on PBS. Grids were floated on drops of 1% (w/v) bovine serum albumin (BSA) in PBS for 10 min to block non-specific binding. All antibodies are protein-A gold were diluted in 1% (w/v) BSA. The primary anti-DNA antibody (MAB030; Chemicon) was

diluted 1:20 and centrifuged at 14,000 rpm for 1 min to remove aggregates. Grids were then incubated on 5 μL drops of primary antibody solution for 30 min at room temperature, the washed in four large drops of PBS for a total of 15 min. Subsequently, grids were incubated with rabbit anti-mouse IgG (Abcam; ab6709) for 30 min.

After washing again on four drops of PBS, grids were incubated for 20 min at room temperature on 5 μL drops of protein A–gold (10nm) (University Medical Center, Utrecht, The Netherlands). Excess protein A–gold was removed by washing the grids on four drops of PBS followed by six drops of double-distilled water. Finally, the grids were contrasted by incubation in 0.3% uranyl acetate in 2% methyl cellulose for 10 min. Excess liquid was carefully removed using Whatman grade 1 filter paper.

Serial cell sectioning. Epon embedded samples from mock- and HSV-1 *d*109-infected HFF cells were serially ultrathin-sectioned and collected sequentially on grids, as described above.

TEM imaging and image analyses. Electron micrographs were taken using a Tecnai G2 Spirit BioTWIN transmission electron microscope equipped with an AMT Nanosprint 43-MKII camera. The resulting transmission electron microscopy micrographs were exported as TIFF files and uploaded into ImageJ (NIH, USA) for analysis. For measurements, the scale was adjusted based on the pixel size in nanometers, indicated on the micrograph (Analyze > Set Scale). The length-measure tool via straight lines (Analyze > Measure) was used to measure distances in zoomed-in areas for higher accuracy in determining the distance between the inner and outer nuclear membranes and nuclear pore diameters. The stretches in the nuclear envelope and nuclear pore numbers were normalized by the total length of the nuclear envelope inspected, determined by freehand line measurements. Scale bar length is indicated in figure legends.

Graphs and statistical analyses. Data were graphed using GraphPad Prism software version 10.2.0. The one-tailed paired Student's t-tests were launched in the Microsoft Excel software

version 16.89.1, and the statistical significance is reached with a p-value lower than or equal to 0.05. The resulting p-values are indicated in the figures and their legends.

Supp.Fig. 1

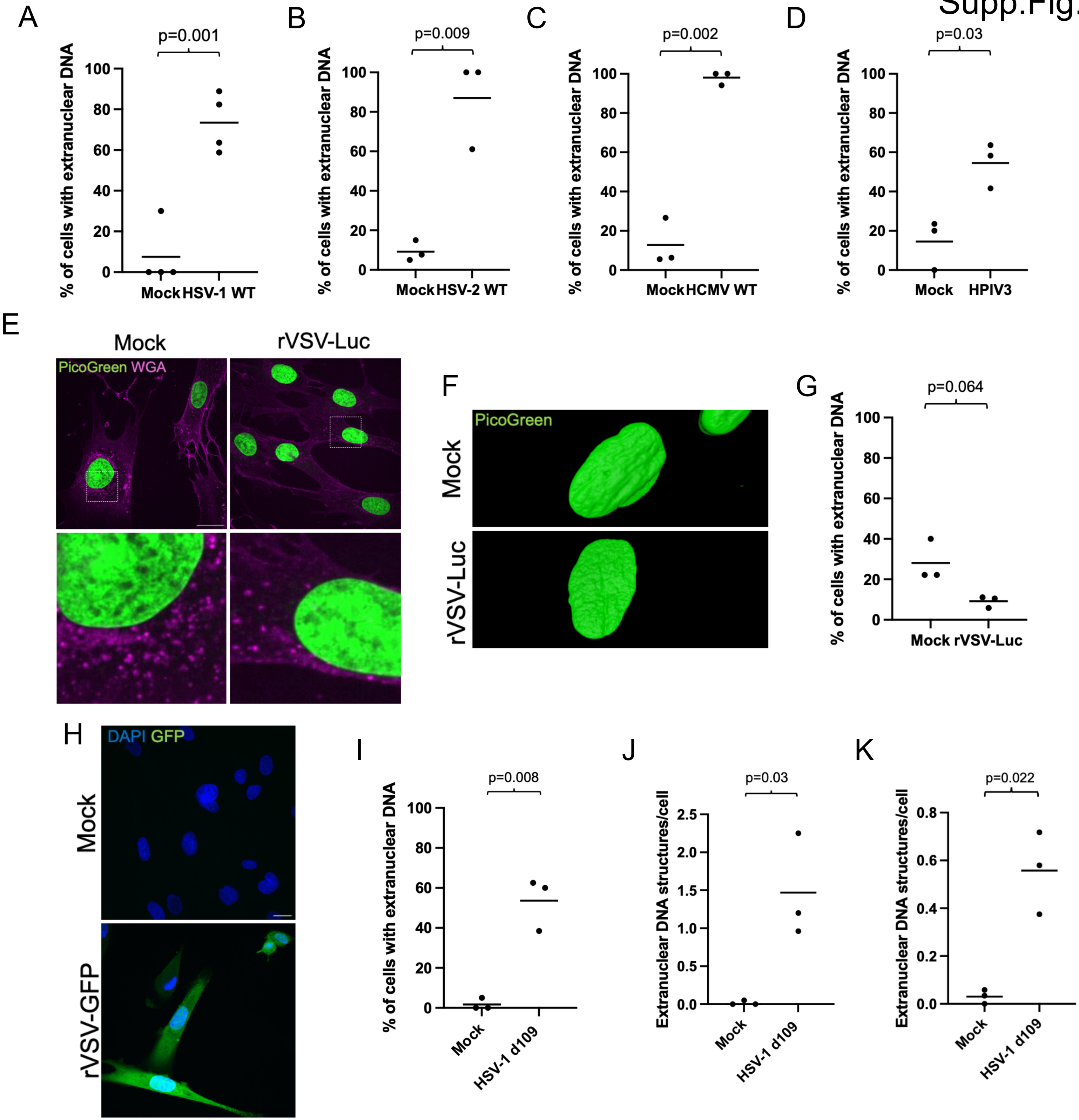

A
p=0.001
% of cells with extranuclear DNA
100
80
60
40
20
0
Mock
HSV-1 WT
B
p=0.009
% of cells with extranuclear DNA
Mock
HSV-2 WT
C
p=0.002
% of cells with extranuclear DNA
Mock
HCMV WT
D
p=0.03
% of cells with extranuclear DNA
Mock
HPIV3
E
Mock
rVSV-Luc
PicoGreen WGA
F
PicoGreen
Mock
rVSV-Luc
G
p=0.064
% of cells with extranuclear DNA
Mock
rVSV-Luc
H
DAPI GFP
Mock
rVSV-GFP
I
p=0.008
% of cells with extranuclear DNA
Mock
HSV-1 d109
J
p=0.03
Extranuclear DNA structures/cell
2.5
2.0
1.5
1.0
0.5
0.0
Mock
HSV-1 d109
K
p=0.022
Extranuclear DNA structures/cell
0.8
0.6
0.4
0.2
0.0
Mock
HSV-1 d109

Supp. Fig. 2

A

Stretches ≥ 60nm/50µm of NE

	Mock	HSV-1 d109
Experiment #1	2.369	8.790
Experiment #2	0.638	3.055

B

Stretches ≥ 100nm/50µm of NE

	Mock	HSV-1 d109
Experiment #1	0.430	2.720
Experiment #2	0	1.309

C

Nuclear pores/50µm of NE

	Mock	HSV-1 d109
Experiment #1	13.356	11.301
Experiment #2	6.810	7.420

D

Nuclear pore diameter

	Mock	HSV-1 d109
Experiment #1	92.62 nm	95.25 nm
Experiment #2	87.99 nm	83.86 nm

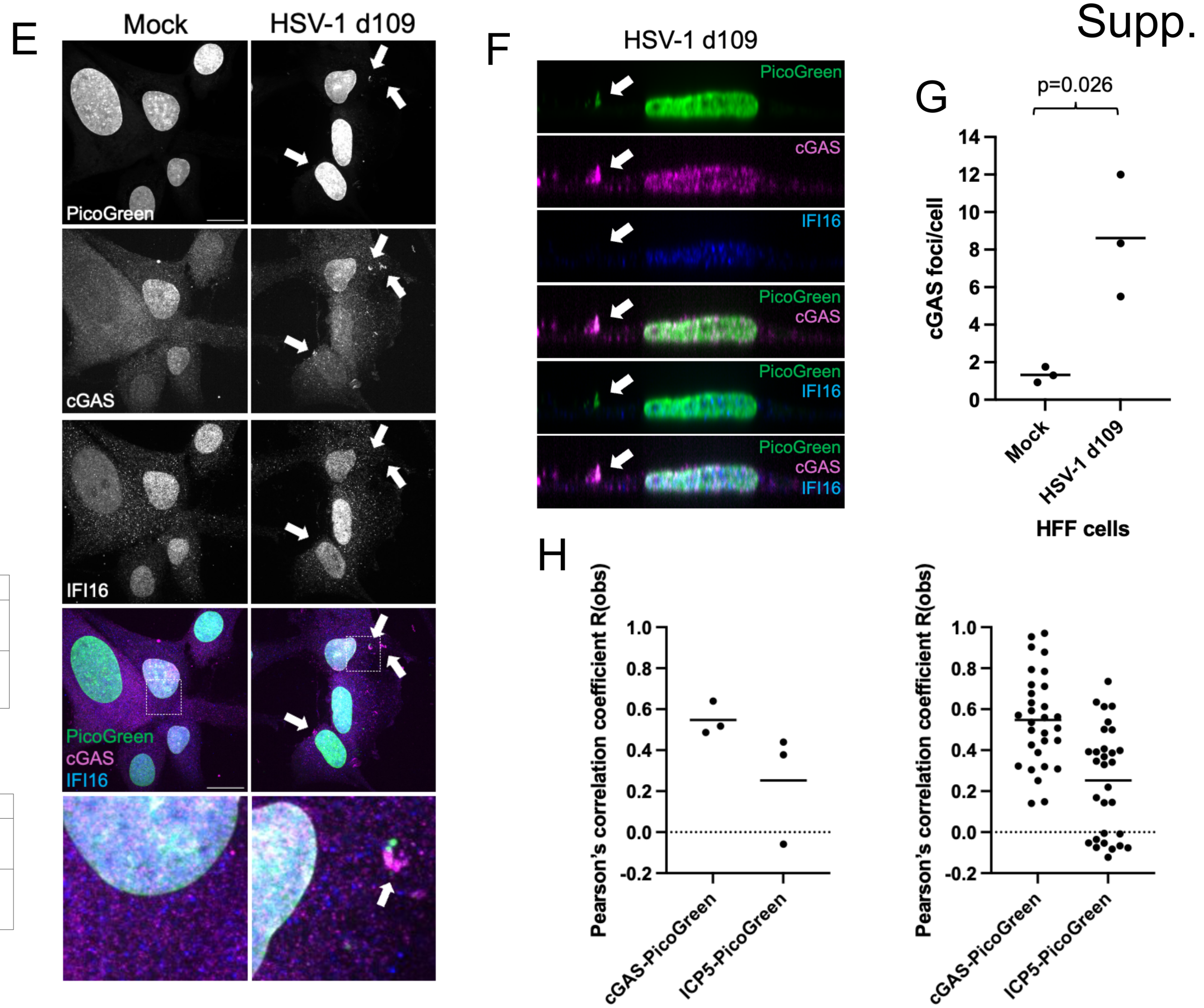

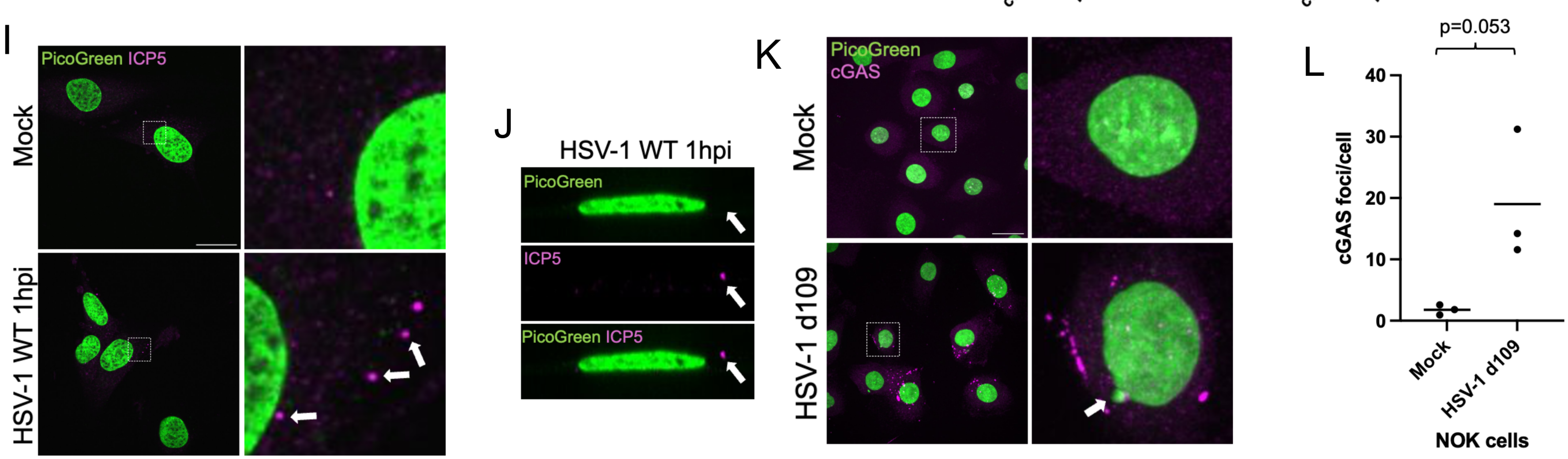

Supp. Fig. 3

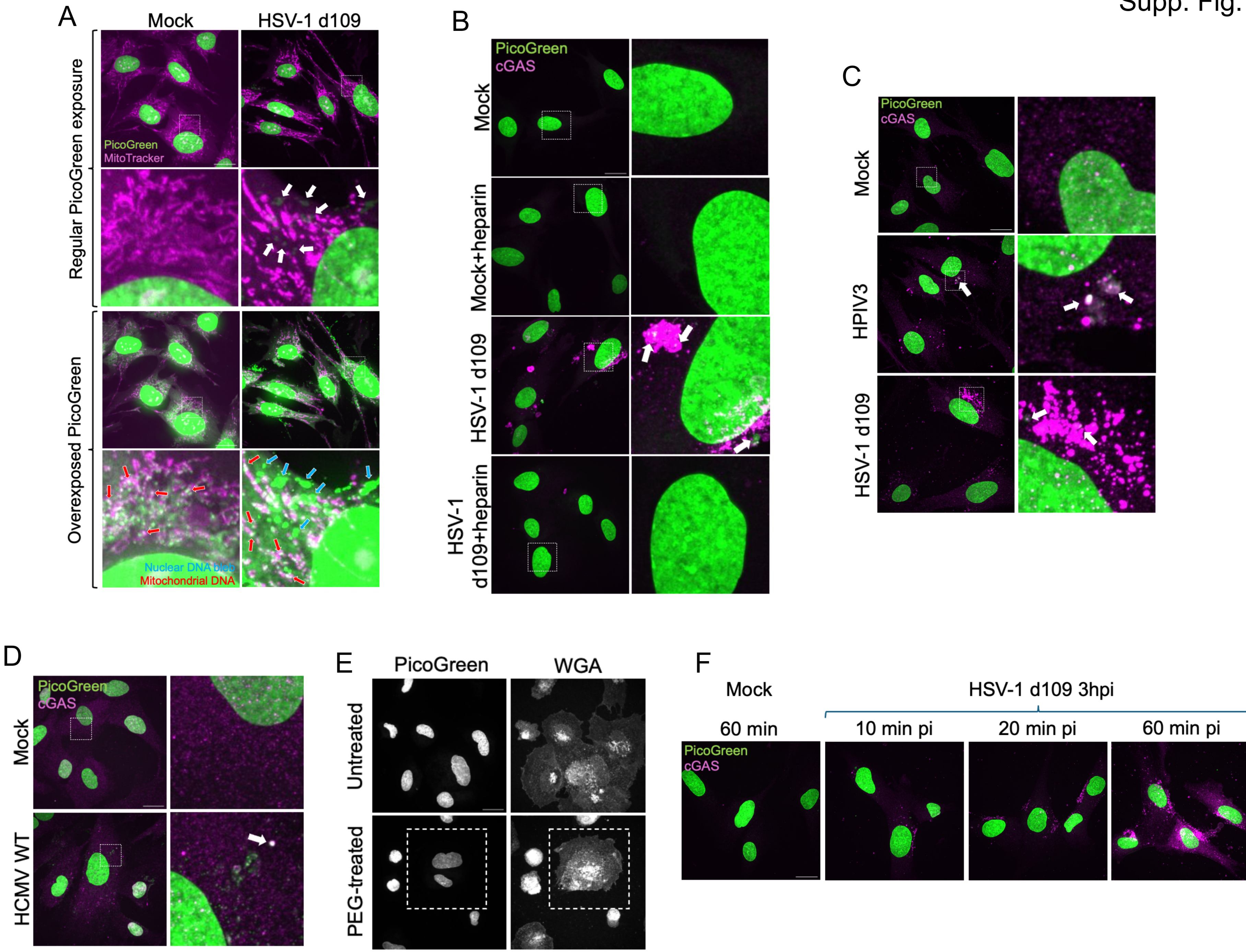

A
Mock
HSV-1 d109
Regular PicoGreen exposure
Overexposed PicoGreen
PicoGreen
MitoTracker
Nuclear DNA blebs
Mitochondrial DNA
B
PicoGreen
cGAS
Mock
Mock+heparin
HSV-1 d109
HSV-1 d109+heparin
C
PicoGreen
cGAS
Mock
HPIV3
HSV-1 d109
D
PicoGreen
cGAS
Mock
HCMV WT
E
PicoGreen
WGA
Untreated
PEG-treated
F
Mock
HSV-1 d109 3hpi
60 min
10 min pi
20 min pi
60 min pi
PicoGreen
cGAS

Figures

Fig. S1. (A) The values shown are individual datapoints and means of the percentages of mock-infected or HSV-1 WT-infected HFF cells positive for cytoplasmic DNA blebs at 1 hpi (n=3 independent experiments) from the experiments in Fig. 1A-C. p = 0.001 using a paired one-tailed Student's t-test. (B) The values shown are the individual datapoints and means (n=3 independent experiments) of the % of mock-infected and HSV-2 WT-infected HFF cells exhibiting extranuclear DNA structures at 1 hpi (MOI = 5 pfu/cell), determined in images from the experiments shown in Figs. 1D-E. p = 0.009 using a paired one-tailed Student's t test. (C) The values shown are individual datapoints and means (n=3 independent experiments) for the % of mock-infected and HCMV WT-infected HFF cells at 1 hpi (MOI = 5 pfu/cell) with extranuclear DNA structures from analysis of images from experiments shown in Figs. 2A-C. p=0.002 from a one-tailed paired Student's t test. (D) The values are shown are individual datapoints and means (n=3 independent experiments) for the % of mock-infected or HPIV3-infected HFF cells with extranuclear DNA structures at 3 hpi (MOI of 1 pfu/cell), analyzed from images from the experiments shown in Figs. 2D-E. p value = 0.03 using a one-tailed paired Student's t test. (E) HFF cells were mock-infected or infected with rVSV-Luc an MOI of 5 and fixed at 3 hpi (n=3) and stained with PicoGreen and Texas-red-conjugated WGA. Green = PicoGreen; magenta = WGA. Inset images show increased magnification. (F) Three-dimensional renderings of PicoGreen signal (green) in mock-infected or rVSV-Luc-infected HFF cells shown from an apical view. (G) The values shown are individual datapoints and means (n=3 independent experiments) for the % of mock-infected or rVSV-Luc-infected HFF cells with cytoplasmic DNA structures at 3 hpi from images from the experiments shown in Supp. Fig. 1E-F. p=0.064 from a paired one-tailed Student's t test. (H) HFF cells were mock-infected or infected with rVSV-GFP at an MOI of 5 pfu/cell, fixed at 24 hpi and stained with DAPI. Green = GFP; blue = DAPI, (n=2 independent experiments). The scale bars in (E) and (H) are 20 µm. (I) The values shown are the individual values and means (n=3 independent experiments) of the percentages of mock-infected or HSV-1 *d*109-infected HFF cells positive for extranuclear DNA structures from the images from the experiments shown in Figs. 3A-B. p =

0.008 using a paired one-tailed Student's t-test. (J) The values shown are the individual datapoints and means (n=3 independent experiments) for the number of extranuclear DNA structures per cell in the Y/Z axes in mock-infected or HSV-1 *d*109-infected HFF cells based on the images from the experiments shown in Figs. 3A-B. p = 0.03 using a paired one-tailed Student's t-test. (K) The values shown are the numbers of discrete extranuclear DNA structures per cell and means (n=3 independent experiments) for mock-infected or HSV-1 *d*109-infected NOK cells, based on images from the experiments shown in Figs. 3G-H. p = 0.022 using a paired one-tailed Student's t-test.

Fig. S2. (A) The values listed are the number of nuclear membrane separations of ≥60nm, and (B) ≥100nm nuclear envelope stretches per 50 µm of nuclear envelope contour and are shown for two independent repeats of the assay. Up to 20 TEM micrographs per condition were evaluated, from the experiments illustrated in Fig. 4A. The length of the nuclear envelope analyzed was for experiment #1: mock = 232.10 µm and HSV-1-*d*109 = 238.90 µm; and for experiment #2: mock = 234.92 µm and HSV-1-*d*109 = 229.11 µm. (C) The values listed are the number of nuclear pores per 50 µm of nuclear envelope, and (D) the means of nuclear pore diameter in mock-infected or HSV-1 *d*109-infected HFF cells at 3 hpi (MOI = 5 pfu/cell). The number of nuclear pores counted in experiment #1 were: mock = 62 and HSV-1-*d*109 = 53; and in experiment #2: mock = 32 and HSV-1-*d*109 = 34, from images from the experiments shown in Fig. 4A. (E) HFF cells were mock-infected or infected with HSV-1 *d*109 at MOI = 5 and fixed at 3 hpi, and stained with anti-IFI16 and -cGAS antibodies. Micrographs are shown as Z-projections. Green = PicoGreen, magenta = cGAS, and blue = IFI16 in merged images. (F) Orthogonal view of HSV-1 *d*109-infected HFF cell from images from the experiment illustrated in (E). PicoGreen = green; cGAS = magenta; IFI16 = blue. (G) The values shown are individual datapoints and means for the number of cGAS foci found in the cytoplasm of mock-infected or HSV-1 *d*109-infected HFF cells at 3hpi (n=3 independent experiments). p = 0.026 using a one-tailed paired Student's t test. (H) The left graph shows the individual values and means (n=3 independent experiments) for Pearson's correlation coefficient R(obs) for cGAS and PicoGreen fluorescent signals in HSV-1 *d*109-infected HFF cells

at 3 hpi, and for HSV-1 ICP5 (input capsids) and PicoGreen signals in HSV-1 WT-infected cells (MOI = 20) fixed at 1 hpi. The right graph shows 30 individual field measurements (ten measurements in each of the three independent repeats). (I) HFF cells were mock-infected or infected with HSV-1 WT (MOI = 20) and fixed at 1 hpi (n=3 independent experiments) and stained with anti-ICP5 antibody and PicoGreen. Z-projections are illustrated. Magenta =HSV-1 ICP5 protein; green = PicoGreen. (J) Orthogonal views from images shown in (I). Green = PicoGreen; magenta = HSV-1 ICP5 protein. White arrows denote input HSV-1 capsids in (I) and (J). (K) NOK cells were mock-infected or infected with HSV-1 *d*109 (MOI = 5) and fixed at 3 hpi (n=3 independent experiments) and stained with anti-cGAS and PicoGreen. Z-projections are represented. Green = PicoGreen; magenta = cGAS. The white arrow denotes a cytoplasmic DNA-cGAS focus. (L) The values shown are individual values and means of the cytoplasmic cGAS foci per cell for mock-infected or HSV-1 *d*109-infected NOK cells at 3 hpi (n=3 independent experiments). p=0.053 using a one-tailed paired Student’s t test. Scale bars in (E), (I), and (K) are 20 μm.

Fig. S3. (A) HFF cells were mock-infected or infected with HSV-1 *d*109 (MOI = 5) and at 2.5 hpi, stained with MitoTracker for 30 min, fixed at 3 hpi, and stained with PicoGreen (n=3 independent experiments). PicoGreen = green and MitoTracker = magenta. Z-projections are represented of images with regular PicoGreen signal exposure or overexposed PicoGreen signal. White arrows denote nuclear DNA blebs in regularly exposed magnified images. In overexposed magnified micrographs, nuclear DNA blebs are denoted with blue arrows and mitochondrial DNA with red arrows. (B) HFF cells were mock-infected or infected with HSV-1*d*109 supernatant virus at an MOI of 5 pfu/cell for 3 hpi in the presence or absence of heparin, which was added to cells 1 h before infection and maintained in the culture during and after virus adsorption. Cells were stained with anti-cGAS antibody and PicoGreen (n=3 independent experiments). Images shown are Z-projections. PicoGreen = green; cGAS = magenta. (C) HFF cells were mock-infected or infected with HSV-1 *d*109 supernatant stocks or HPIV3 F K108 eGFP virus (HPIV3) at MOI = 1, fixed at 3 hpi (n=3 independent experiments), and stained with anti-cGAS antibody and

PicoGreen. The confocal microscopy images presented correspond to Z-projections. Inset images show increased magnifications of cytoplasmic DNA-cGAS foci. Magenta = cGAS; green = PicoGreen. (D) HFF cells were mock-infected or infected with HCMV strain AD169 (HCMV WT) supernatant stock with an MOI of 5 pfu/cell, fixed at 3 hpi, and stained with anti-cGAS antibody and PicoGreem (n=3 independent experiments). Z-projections are shown. PicoGreen = green and cGAS = magenta. White arrows denote cytoplasmic DNA-cGAS foci in (B), (C), and (D). (E) HFF cells incubated with polyethylene glycol (PEG) 3350 or control buffer, fixed at 3 h, and stained with PicoGreen (green) and Texas red-conjugated WGA (magenta) (n=3 independent experiments). Multinucleated cells were identified as those with two or more nuclei without a discontinuity in the plasma membrane perimeter defined by WGA labeling. They are denoted with a dotted line in Z-projection micrographs. (F) An MOI = 5 pfu/cell of HSV-1 *d*109 supernatant stock was bound or not to HFF cells at 4ºC for 1 h and then, cells mock-infected and infected cells were placed at 37ºC. HSV-1 *d*109-infected cells were treated with citric acid buffer pH 3.5 for 1 min at room temperature to inactivate unentered HSV-1 virions from cell surface at 10, 20 or 60 min postinfection (pi), before incubation of cells in fresh HFF medium at 37ºC. Mock-infected cells were treated with citric acid buffer at 60 min pi. Cells were fixed at 3 hpi and stained with PicoGreen and anti-cGAS antibody (n=3 independent repeats). PicoGreen = green, cGAS = magenta in Z-projection micrographs. DNA-cGAS foci are denoted with white arrows. Scale bar is 20 µm in (A)-(F).

SI References

1. **Sodroski CN, Knipe DM.** 2023. Nuclear interferon-stimulated gene product maintains heterochromatin on the herpes simplex viral genome to limit lytic infection. Proc Natl Acad Sci U S A **120:**e2310996120.

2. **Piboonniyom SO, Duensing S, Swilling NW, Hasskarl J, Hinds PW, Münger K.** 2003. Abrogation of the retinoblastoma tumor suppressor checkpoint during keratinocyte

immortalization is not sufficient for induction of centrosome-mediated genomic instability. Cancer Res **63:**476-483.

3. **Samaniego LA, Wu N, DeLuca NA.** 1997. The herpes simplex virus immediate-early protein ICP0 affects transcription from the viral genome and infected-cell survival in the absence of ICP4 and ICP27. J Virol **71:**4614-4625.
4. **Cabral JM, Oh HS, Knipe DM.** 2018. ATRX promotes maintenance of herpes simplex virus heterochromatin during chromatin stress. eLife **7:**e40228.
5. **Smith KO.** 1964. Relationship between the envelope and the infectivity of herpes simplex virus. Proc Soc Exp Biol Med **115:**814-816.
6. **Colgrove RC, Liu X, Griffiths A, Raja P, Deluca NA, Newman RM, Coen DM, Knipe DM.** 2016. History and genomic sequence analysis of the herpes simplex virus 1 KOS and KOS1.1 sub-strains. Virology **487:**215-221.
7. **Conwell SE, White AE, Harper JW, Knipe DM.** 2015. Identification of TRIM27 as a novel degradation target of herpes simplex virus 1 ICP0. J Virol **89:**220-229.
8. **Cai WZ, Schaffer PA.** 1989. Herpes simplex virus type 1 ICP0 plays a critical role in the de novo synthesis of infectious virus following transfection of viral DNA. J Virol **63:**4579-4589.
9. **Samaniego LA, Neiderhiser L, DeLuca NA.** 1998. Persistence and expression of the herpes simplex virus genome in the absence of immediate-early proteins. J Virol **72:**3307-3320.
10. **Ejercito PM, Kieff ED, Roizman B.** 1968. Characterization of herpes simplex virus strains differing in their effect on social behavior of infected cells. J Gen Virol **2:**357-364.
11. **Zhou C, Knipe DM.** Manuscript in preparation.
12. **Elek SD, Stern H.** 1974. Development of a vaccine against mental retardation caused by cytomegalovirus infection in utero. Lancet **1:**1-5.
13. **Stearns K, Lampe G, Hanan R, Marcink T, Niewiesk S, Sternberg SH, Greninger AL, Porotto M, Moscona A.** 2024. Human parainfluenza virus 3 field strains undergo extracellular fusion protein cleavage to activate entry. mBio **15:**e0232724.

14. **Cureton DK, Massol RH, Saffarian S, Kirchhausen TL, Whelan SP.** 2009. Vesicular stomatitis virus enters cells through vesicles incompletely coated with clathrin that depend upon actin for internalization. PLoS Pathog **5:**e1000394.

15. **Whelan SP, Barr JN, Wertz GW.** 2000. Identification of a minimal size requirement for termination of vesicular stomatitis virus mRNA: implications for the mechanism of transcription. J Virol **74:**8268-8276.

16. **Whelan SP, Ball LA, Barr JN, Wertz GT.** 1995. Efficient recovery of infectious vesicular stomatitis virus entirely from cDNA clones. Proc Natl Acad Sci U S A **92:**8388-8392.

17. **Orzalli MH, DeLuca NA, Knipe DM.** 2012. Nuclear IFI16 induction of IRF-3 signaling during herpesviral infection and degradation of IFI16 by the viral ICP0 protein. Proc Natl Acad Sci U S A **109:**E3008-3017.

18. **Morrison LA, Knipe DM.** 1996. Mechanisms of immunization with a replication-defective mutant of herpes simplex virus 1. Virology **220:**402-413.

19. **Chang L, Godinez WJ, Kim IH, Tektonidis M, de Lanerolle P, Eils R, Rohr K, Knipe DM.** 2011. Herpesviral replication compartments move and coalesce at nuclear speckles to enhance export of viral late mRNA. Proc Natl Acad Sci U S A **108:**E136-144.

20. **Mertens ME, Knipe DM.** 2021. Herpes Simplex Virus 1 Manipulates Host Cell Antiviral and Proviral DNA Damage Responses. mBio **12:**e03552-03520.

21. **Orzalli MH, Broekema NM, Diner BA, Hancks DC, Elde NC, Cristea IM, Knipe DM.** 2015. cGAS-mediated stabilization of IFI16 promotes innate signaling during herpes simplex virus infection. Proc Natl Acad Sci U S A **112:**E1773-1781.

22. **Zhou X, Platt JL.** 2011. Molecular and Cellular Mechanisms of Mammalian Cell Fusion, p 33-64. *In* Dittmar T, Zänker KS (ed), Cell Fusion in Health and Disease. Springer Netherlands, Dordrecht.

23. **Griffiths G.** 1993. Fine Structure Immunocytochemistry. Springer-Verlag, Berlin.
